# The Protein-Protein Interaction tasks of BioCreative III: classification/ranking of articles and linking bio-ontology concepts to full text

**DOI:** 10.1186/1471-2105-12-S8-S3

**Published:** 2011-10-03

**Authors:** Martin Krallinger, Miguel Vazquez, Florian Leitner, David Salgado, Andrew Chatr-aryamontri, Andrew Winter, Livia Perfetto, Leonardo Briganti, Luana Licata, Marta Iannuccelli, Luisa Castagnoli, Gianni Cesareni, Mike Tyers, Gerold Schneider, Fabio Rinaldi, Robert Leaman, Graciela Gonzalez, Sergio Matos, Sun Kim, W John Wilbur, Luis Rocha, Hagit Shatkay, Ashish V Tendulkar, Shashank Agarwal, Feifan Liu, Xinglong Wang, Rafal Rak, Keith Noto, Charles Elkan, Zhiyong Lu, Rezarta Islamaj Dogan, Jean-Fred Fontaine, Miguel A Andrade-Navarro, Alfonso Valencia

**Affiliations:** 1 Structural Biology and BioComputing Programme, Spanish National Cancer Research Centre (CNIO), Madrid, Spain; 2 Australian Regenerative Medicine Institute, Monash University, Australia; 3 School of Biological Sciences, University of Edinburgh, Edinburgh, UK; 4 Department of Biology, University of Rome Tor Vergata, Rome, Italy; 5 IRCSS, Fondazione Santa Lucia, Rome, Italy; 6 Institute of Computational Linguistics, University of Zurich, Zurich, Switzerland; 7 School of Computing, Informatics and Decision Systems Engineering, Arizona State University, Tempe, Arizona, USA; 8 Department of Biomedical Informatics, Arizona State University, Tempe, Arizona, USA; 9 Institute of Electronics and Telematics Engineering of Aveiro, University of Aveiro Campus Universitario de Santiago, 3810-193 Aveiro, Portugal; 10 National Center for Biotechnology Information (NCBI), 8600 Rockville Pike, Bethesda, Maryland, 20894, USA; 11 School of Informatics and Computing, Indiana University, 919 E. 10th St Bloomington IN, 47408, USA; 12 Department of Computer and Information Sciences, University of Delaware, Newark, DE 19716, USA; 13 Department of Computer Science and Engineering, IIT Madras, Chennai-600 036, India; 14 Medical Informatics, University of Wisconsin-Milwaukee, Milwaukee, Wisconsin, USA; 15 National Centre for Text Mining and School of Computer Science, University of Manchester, Manchester, UK; 16 Department of Computer Science, Tufts University, 161 College Ave, Medford, MA 02155, USA; 17 Department of Computer Science and Engineering, University of California, San Diego, 9500 Gilman Drive, La Jolla, CA 92093, USA; 18 Computational Biology and Data Mining Group, Max-Delbrück-Centrum für Molekulare Medizin, Robert-Rössle-Str. 10, 13125 Berlin, Germany

## Abstract

**Background:**

Determining usefulness of biomedical text mining systems requires realistic task definition and data selection criteria without artificial constraints, measuring performance aspects that go beyond traditional metrics. The BioCreative III Protein-Protein Interaction (PPI) tasks were motivated by such considerations, trying to address aspects including how the end user would oversee the generated output, for instance by providing ranked results, textual evidence for human interpretation or measuring time savings by using automated systems. Detecting articles describing complex biological events like PPIs was addressed in the Article Classification Task (ACT), where participants were asked to implement tools for detecting PPI-describing abstracts. Therefore the BCIII-ACT corpus was provided, which includes a training, development and test set of over 12,000 PPI relevant and non-relevant PubMed abstracts labeled manually by domain experts and recording also the human classification times. The Interaction Method Task (IMT) went beyond abstracts and required mining for associations between more than 3,500 full text articles and interaction detection method ontology concepts that had been applied to detect the PPIs reported in them.

**Results:**

A total of 11 teams participated in at least one of the two PPI tasks (10 in ACT and 8 in the IMT) and a total of 62 persons were involved either as participants or in preparing data sets/evaluating these tasks. Per task, each team was allowed to submit five runs offline and another five online via the BioCreative Meta-Server. From the 52 runs submitted for the ACT, the highest Matthew's Correlation Coefficient (MCC) score measured was 0.55 at an accuracy of 89% and the best AUC iP/R was 68%. Most ACT teams explored machine learning methods, some of them also used lexical resources like MeSH terms, PSI-MI concepts or particular lists of verbs and nouns, some integrated NER approaches. For the IMT, a total of 42 runs were evaluated by comparing systems against manually generated annotations done by curators from the BioGRID and MINT databases. The highest AUC iP/R achieved by any run was 53%, the best MCC score 0.55. In case of competitive systems with an acceptable recall (above 35%) the macro-averaged precision ranged between 50% and 80%, with a maximum F-Score of 55%.

**Conclusions:**

The results of the ACT task of BioCreative III indicate that classification of large unbalanced article collections reflecting the real class imbalance is still challenging. Nevertheless, text-mining tools that report ranked lists of relevant articles for manual selection can potentially reduce the time needed to identify half of the relevant articles to less than 1/4 of the time when compared to unranked results. Detecting associations between full text articles and interaction detection method PSI-MI terms (IMT) is more difficult than might be anticipated. This is due to the variability of method term mentions, errors resulting from pre-processing of articles provided as PDF files, and the heterogeneity and different granularity of method term concepts encountered in the ontology. However, combining the sophisticated techniques developed by the participants with supporting evidence strings derived from the articles for human interpretation could result in practical modules for biological annotation workflows.

## Background

Providing access to information relevant to protein interaction characterizations is of great importance both in the field of experimental biology as well as from the perspective of systems biology and bioinformatics analysis. In case of experimental biology, a range of different methodologies have been developed to detect protein interactions, showing different degrees of reliability or underlying properties of the interactions. To enable systematic analysis of interaction networks, the construction of interaction databases such as BioGRID [1], MINT [2], or IntAct [3] - which store interaction annotations in form of well structured database records using standard formats - is essential. These databases rely on specifically trained human curators who manually extract protein interactions from scientific articles, making use of controlled vocabulary terms (covering interaction detection experiments) from the PSI-MI ontology to qualify each interaction [4]. Through such a structured vocabulary, users are able to understand the general conditions underlying a particular interaction annotation, which can be used for selecting customized interaction networks based on experimental qualifiers. Manually generating literature annotations is very time consuming and there are increasing concerns that such approaches are only able to cope with a small fraction of the relevant information published in the growing amount of articles [5,6]. This has motivated a significant amount of research in the biomedical text mining community devoted to the systematic extraction of protein-protein interaction (PPI) information from scientific articles, mainly focusing on the detection of binary associations [7-13]. The detection of interacting protein pairs using information extraction and literature mining techniques has already been addressed carefully in both the BioCreative II and II.5 challenges [14,15]. In order to determine current bottlenecks in literature curation and understand where text mining can actually be of practical use, it is important to formalize the curation using annotation workflows [16]. In case of protein interaction annotation two important steps consist of the initial selection of relevant articles and the association of these to experimental interaction methods. When associations between proteins are retrieved automatically from the literature, determining the corresponding experimental qualifier is crucial in order to characterize whether it actually corresponds to an experimentally validated physical interaction or constitutes general background knowledge or even some other sort of relation (e.g. genetic/gene regulation interaction, indirect association or phenotypic relationship).

### Classification/ranking of articles: Article Classification Task - ACT

Classification and ranking of articles according to a particular topic of interest, such as protein-protein interaction (PPI) is not only useful to improve subsequent bio-entity recognition and relation extraction approaches, but is labeled in itself important for more general purposes [17]. It has been used for prioritizing articles for manual literature curation and can improve the selection of interaction characterizations described in articles mentioning a particular protein or term of interest [18]. This motivated the construction of automated systems able to classify and rank large sets of potentially relevant abstracts for subsequent manual inspection [19-22]. Choosing relevant articles for manual examination in order to derive biological annotations is a general step across almost all biological annotation databases [23]. Potentially relevant collections of articles are often represented by lists of PubMed entries resulting from keyword searches or in lists of recent articles from journals of interest. For complex biological events like PPIs, simple keyword queries are often inefficient in detecting relevant articles. For instance the following evidence sentence for an interaction event does not contain commonly used interaction terms like bind or interact: 'A complex containing Mus81p and Rad54p was identified in immunoprecipitation experiments' (PMID:10905349). Using the term 'complex' as a query for interaction articles would retrieve over 700 thousand PubMed hits, most of them not relevant for interactions. On the other hand, detecting patterns used to express protein interactions like: '*complex containing PROTEIN and PROTEIN' *together with a machine learning system that detects that 'immunoprecipitation' is a feature of PPI articles would be able to score such a record as protein interaction relevant.

The evaluation of article retrieval algorithms for annotation databases has been studied in detail in the context of the former TREC Genomics tracks [24-27], and several BioCreative challenges, namely BioCreative II [14] and II.5 [15]. In case of Biocreative III, the purpose of the (Interaction) Article Classification Task was to promote the development of automated systems that are able to classify articles as relevant for protein-protein interaction (PPI) database curation efforts. The resulting text mining tools should be able to simplify the identification of relevant articles for a range of journals known to publish protein interaction reports.

These modifications included the use of PubMed abstracts as opposed to full text articles as used in the previous BioCreative II.5, as they do not have restrictions in terms of availability. A large range of journals considered as relevant by biological databases have been utilized, avoiding inclusion of those not being used for curation. For this task, large manually classified training, development and test data sets have been prepared to facilitate the implementation of supervised learning methods and to carry out a statistical sound evaluation. Additionally, we considered a publication time range selection criteria to focus on recent articles and provide a more coherent data collection. Finally the sampling used for articles in the development and test sets reflects a more realistic class imbalance (proportion of relevant and non-relevant articles) encountered for these journals. The Gold Standard annotations were generated by domain experts through inspection of a randomly sampled set of abstracts following classification guidelines which were refined during several rounds of classification based on the feedback of the BioGRID and MINT database curators (see additional file 1 for annotation guidelines). Preparing these guidelines required examining a substantial collection of initial example records in order to specifically describe aspects for considering a particular record as PPI relevant. To support this, a set of interaction evidence passages was analyzed, and criteria for non-relevant articles were formalized. Additional example cases for both relevant and non-relevant records had to be discussed with domain experts and feedback from PPI database curators was requested.

### Linking bio-ontology concepts to full text: Interaction Method Task - IMT

In the domain of biomedical sciences, the experimental context is crucial for the interpretation of biological assertions as well as to determine the reliability of a given biological finding [28]. An important aspect for the annotation of protein interactions is to identify the experimental techniques ('interaction detection methods') described in an article to support the interactions [29]. Annotation of experimental techniques or 'evidence' is also common with other annotation efforts, such as the Gene Ontology Annotations (GOA; in the form of evidence codes) [30]. Knowing the experimental method that provided the evidence for an interaction serves as 'credibility' or likelihood indicator that the reported interaction actually occurs in a living organism (*in vivo*) or cell culture (*in vitro*). These types of text classification tasks are based on associating standardized terms from a controlled vocabulary to the text in question. In the case of protein-protein interaction annotations, efforts have been made to develop a controlled vocabulary ('ontology') about interaction detection methods in order to standardize the terminology serving as experimental evidence support. Database curators spend a considerable amount of time determining which experimental evidence supports interaction pairs described in articles [31]. A relevant work in this respect was the implementation of a system for detecting experimental techniques in biomedical articles by Oberoi and colleagues [32]. Also the construction of a text mining system with a particular focus on interaction detection methods using statistical inference techniques has been explored recently [33], motivated by the Interaction Method Task of the BioCreative II challenge [14], where two different teams provided results [34,35]. Even the use of a particular list of affixes corresponding to experimental tags used for labeling interactor proteins (PPI affix dictionary - PPIAD) has been analyzed [36]. For instance the affixes 'GST-' and 'TAP-' show an association to the interaction detection methods 'pull down' and 'tandem affinity purification' respectively. For BioCreative III, participants were asked to provide a list of interaction detection method identifiers for a set of full-text articles, ordered by their likelihood of having been used to detect the PPIs described in each article. These identifiers belong to the set of standardized experimental interaction detection method terms provided by the PSI-MI ontology. The aim of the evaluation was to estimate the facilitation of database curation efforts by providing a list of the most likely PSI-MI identifiers and possibly increase a curator's performance.

## Methods and data

### Data preparation

One important aspect of the Biocreative efforts is to provide Gold Standard data collections that can be used by system developers to implement and evaluate their methods during the challenges as well as afterwards. Preparing large enough and representative data samples is often difficult for the text mining community as it requires the availability of dedicated domain experts to carry out the annotation process. In order to offer practically relevant task data sets, we have collaborated with experienced open access database curators from the BioGRID and MINT databases as well as domain experts from commercial database developers (Reverse Informatics) especially trained for this task (see supplementary material section, additional file 1). All data collections used for the PPI task are available at: http://www.biocreative.org/resources/corpora/biocreative-iii-corpus/.

#### ACT data

Three data collections have been prepared for ACT participants; an overview of the main characteristics of these data collections is illustrated in table 1. The training set (TR) consists of a balanced collection of 2,280 recent articles classified through manual inspection using the MyMiner interface ([37] -http://myminer.armi.monash.edu.au), divided into PPI relevant and non-relevant articles. The annotation guidelines for this task were refined iteratively based on the feedback from both annotation databases and specially trained domain experts. A subset of the PPI relevant records in the training set were selected articles already annotated by PPI databases. In order to improve the practical relevance of the ACT task setting, we prepared the development (DE) and test (TE) set taking into account PPI annotation relevant journals based on the current content of collaborating PPI databases. Random samples of abstracts from these journals were taken to generate a development set of 4,000 abstracts in total and a test set of 6,000 abstracts, i.e., these two disjoint sets were drawn from the same sample collection. Records from these data collections were manually revised, providing a class label for each record along with the manual classification time. For this purpose the MyMiner tool 'File Labeling' system was used. This system improves manual classification time and allows visualization of positive and negative highlighted areas. Highlights permit users to spot words - or parts of words - related to their topics of interest. Annotators can stop the curation process at any moment by saving the classified document. The time spent to decide if a document is related or not to a topic is recorded. Using this MyMiner file labeling tool - as compared to simple unassisted baseline classification - can reduce classification time by a factor of 10, without altering the classification quality. This reduction had been determined using a pilot study based on 3 annotators using 400 article abstracts. For labeling purposes, the segregation into short notepads of 100 records each was carried out, and annotators were requested to have enough pauses during the process to avoid mislabeling due to fatigue.

**Table 1:** ACT data overview

Data set	Tot. articles	PPI	not PPI	Perc. PPI	Years	Journals
Training	2,280	1,140	1,140	50%	2007-2010	118

Development	4,000	682	3,318	17.05%	2009-2010	113

Test	6,000	910	5,090	15.00%	2009-2010	112

Total	12,280	2,732	9,548	-	2007-2010	121

Overview of the data collections provided for ACT

#### IMT data

In order to associate interaction detection methods to articles, full text papers need to be considered, as detailed experimental characterizations are usually not summarized in abstracts. We provided participants with three basic data collections for this task (see table 2 for a general overview). General data requirements included: (1) The article should be annotated with valid interaction detection methods by a trained PPI database curator following the PSI-MI standards. (2) Only articles from journals belonging to publishers that granted the necessary permissions to the organizers of this challenge could be included.

**Table 2:** IMT data overview

Data set	Tot. articles	Annotations	PSI-MI IDs	IDs/article	Years	Journals
Training	2,003	4,348	86	2.17	2006-2010	87

Development	587	1,316	71	2.24	2006-2010	17

Test	223	528	46	2.36	2008-2010	9

Overview of the data collections provided for IMT

**Figure 1:**
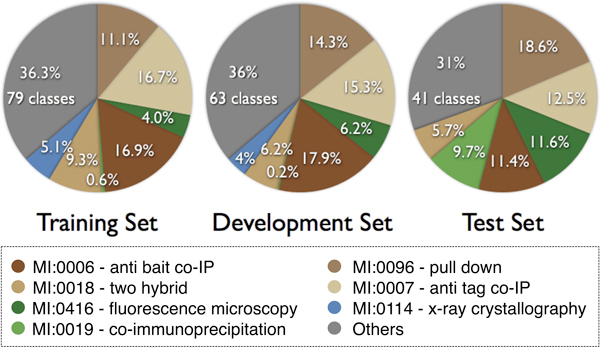
**IMT data set class distribution**. Pie charts illustrating the most frequent methods encountered in the three IMT data collections. Classes are ordered by their frequency in the test set. The most frequent training and development set classes are shown in shades of brown that in total contribute more than 50% of all class assignments in those two sets. In blue is class MI:0114 (x-ray crystallography) that is not frequent in the test set. In green are classes that are significantly more frequent in the test set than the others. MI:0018 (two hybrid) and MI:0114 are frequent in the training and development set, while MI:0416 (fluorescence microscopy) and MI:0019 (coimmunoprecipitation) are frequent in the test set.

During an initial analysis, we identified candidate journals that were the source of curated PPI annotations. (3) Articles should be available as PDF files that can be converted to plain text. Participants were supplied with 2,003 articles as training set and received an additional 587 articles as development set shortly before the test phase. The annotations of these two collections were derived from annotations of PSI-MI compliant databases. Obsolete annotations were remapped and a set of overly general terms that are not considered as useful by annotation databases were filtered out. The final collection of allowed interaction detection method terms contained 115 terms. Team predictions were evaluated using a test of 305 unseen publications, 223 of which were annotation-relevant articles. Both the training and test sets had a highly distorted representation of the 115 possible method detection terms, with only 4 methods representing roughly half of all annotations made on the articles. These 4 high-frequency terms are (from most to least frequent): 'anti bait coimmunoprecipitation', 'anti tag coimmunoprecipitation' (these two represent 1/3 of all annotations), 'pull down', and 'two hybrid'. Figure 1 shows the distribution of interaction methods in the different data collections.

### Evaluation metrics and result structure

The general evaluation setting of the PPI task was to provide successively labeled data collections (first training and then development sets) to participating systems (see table 3 for an overview on participant teams) together with the corresponding evaluation software which is available at: http://www.biocreative.org/media/store/files/2010/bc_evaluation-2.3.1.tar.gz. During this initial phase, teams could implement their systems and improve them using the data sets provided. In case some difficulties or unclear aspects were encountered, registered participants could obtain feedback either by directly contacting the organizers or through a special BioCreative mailing list. During the test phase, teams retrieved a data set for which the labels were held back. They had to provide predictions in a specific format. These predictions were evaluated by comparing them to manual annotations. The actual evaluation scores used are similar to metrics applied during BC II.5, and included Accuracy, Sensitivity (Recall), Specificity, F-Score, Matthews Correlation Coefficient (MCC; the most stable of these evaluation function on unbalanced sets) and the area under the (interpolated) Precision/Recall curve (AUC iP/R). A detailed description of these scores is provided in the BC II.5 overview paper [15].

**Table 3:** PPI task participating teams

TeamId	Leader	Institution	Country	ACT	IMT	URL
65	Fabio Rinaldi	University of Zurich	Switzerland	5	5	[75]

69	Robert Leaman	Arizona State University	USA	0	5	[76]

70	Sergio Matos	Universidade de Aveiro, IEETA	Portugal	5	5	-

73	W John Wilbur	NCBI	USA	5	0	[77]

81	Luis Rocha	Indiana University	USA	10	5	-

88	Ashish Tendulkar	IIT Madras	India	2	2	[78,79]

89	Shashank Agarwal	University of Wisconsin-Milwaukee	USA	10	10	[80,81]

90	Xinglong Wang	National Centre for Text Mining	UK	5	5	-

92	Keith Noto	Tufts University	USA	1	0	[66]

100	Zhiyong Lu	NCBI	USA	4	5	-

104	Jean-Fred Fontaine	Max Delbrück Center	Germany	5	0	[82]

Overview of teams that participated in the PPI tasks and availability of resulting systems. The numbers in the column ACT and IMT correspond to the number of submitted runs by that team.

In case of the ACT, for each article, participants had to return a Boolean value (true/false) regarding its relevance for PPI curation, together with a confidence score for this classification (in the range (0,1]), and the overall (unique) rank of the article in the whole set of articles with respect to its PPI relevance. The main utility measure of a system - i.e., the primary evaluation score for this tasks - is based on measuring a system's ability to provide the best possible ranked list of relevant abstracts, sorted from the most relevant (i.e., highest ranked article that is classified as true) to the most irrelevant article (i.e., highest ranked article classified as false). To this end, the area under the (interpolated) Precision/Recall curve is measured (AUC iP/R score) by using the results' ranking. We also added the F-Scores for comparison to the BioCreative II results.

In case of the IMT, for each article, participants had to return zero or more PSI-MI detection method term identifiers, and for each term annotation they had to provide a confidence score (in the range (0,1]), and an overall (unique) rank for each term annotated on an article, from the most to the least relevant. In addition, participants were asked to return the most decisive evidence text passage that gave rise to their annotation - data useful for human interpretation. The primary metric used for the IMT was based on the average per-article annotation performance (macro-averaging) given its ranking: The area under the (interpolated) Precision/Recall curve was measured (AUC iP/R) by averaging the AUC from the individual scores on each article. (For more general information evaluation metric calculations please refer to the additional materials section).

## Results

### ACT

#### ACT inter-annotator and manual classification time analysis

A set of 649 articles has been annotated by contracted domain experts as well as MINT curators (development set, DE: 360; test set, TE: 289 articles), or by curators from BioGrid (DE: 365; TE: 284 articles), and 200 of these double-annotated articles were annotated by one representative of all three groups - domain expert, MINT, and BioGrid. Four annotations with curation times significantly over 10 minutes were discarded as outliers in the following analysis (PMIDs: 19517012, 19515822, 19718269, and 19774229) - the most extreme outlier had a recorded annotation time of more than six hours.

**Figure 2:**
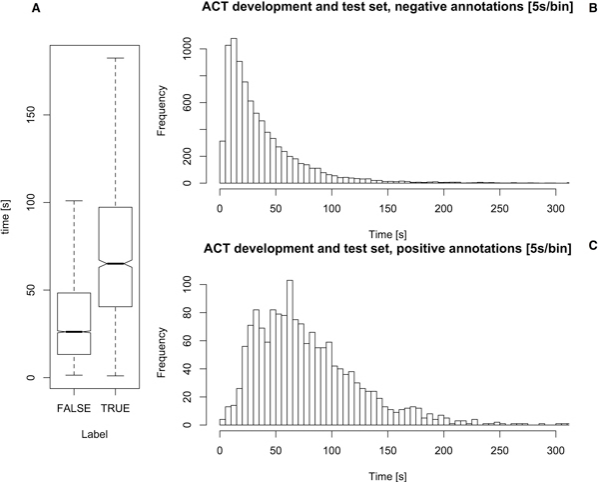
**ACT manual classification time per class**. (**A**) Box plot of the manual classification time distribution. (**B**) ACT development and test set annotation time histogram for negative (non-PPI) abstracts and (**C**) for positive (PPI relevant) abstracts.

**Figure 3:**
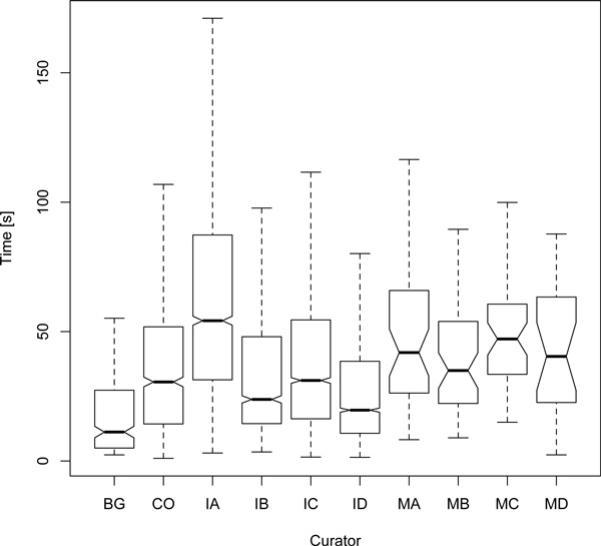
**ACT manual classification time per curator**. Box plot of manual classification time spent by each individual curator. The labels correspond to: four expert curators, ordered by experience (lowest = IA, highest = ID), a CNIO annotator (CO), a BioGRID curator (BG), and four MINT curators (MA-MD).

Over the entire set, the average curation time was 43 sec/article (median = 31; s.d. = 42). Splitting this combined set into positive and negative labels made by the annotators uncovers a large difference in the mean: 75 sec for articles that curators tagged as positive, and 37 sec for the negative case, a ratio of roughly 2:1 for curating negative articles (see Figure 2). The difference is significant using Wilcoxon rank-sum test [38] for non-normal distributions (*p *= 2.2e - 16). Additionally, a nonparametric confidence interval (at 95%) for the differences in locations was computed [39], at 38.42 ± 1.85 sec (and matches the difference in means). This might be an indicator that annotation time of positive articles is significantly longer (double) than for negative articles, even though it is only based on the curation time data of ten individual curators (see Figure 3). To ensure this ratio is persistent across all curators, the median time spent curating positive articles was divided by the median time spent by the curator to label negative articles (the median time was used to reduce the effect of outliers). The ratios were found as follows: IA = 1.7, IB = 4.1, IC = 2.8, ID = 3.1, BG = 3.9, MA = 1.0, MB = 0.9, MC = 1.0, MD = 1.7, CO = 1.6. Overall, curation time for positive articles tended to be about twice as long as for negative articles, but this ratio was quite curator-dependent. The expert curators are ordered by experience (IA: domain expert specially trained for this task, IC and IB: domain experts with a previous experience of 6 months on similar tasks and ID: domain expert with over 3 years experience on similar tasks). Expert curator IA took more time in reading the complete abstract whereas the more experienced curators (IB, IC, ID) could read faster and it was easy for them to identify the most irrelevant abstracts (e.g from other fields like chemistry, ecosystem studies or database descriptions). The MINT and BioGrid curators are all professional PPI database annotators with a higher level of experience. The CNIO annotator (CO) has a degree of training equivalent to the expert curators IB and IC. Thus, there seems to be a correlation between curator experience and this proportion. More generally, an overall tendency towards more curation time needed for positive articles by less experienced curators can be observed, although the exact property of this behavior will be hard to capture numerically.

Second, we investigated the correlation of article curation time and article word (token) length. A Pearson's correlation test was carried out for each curator. For the expert curators and the CNIO annotator (CO), there was a high correlation between the two variables (IA: t = 14.5, df = 2594, p-value < 2.2e-16; IB: t = 7.4, df = 1392, p-value = 2.6e-13; IC: t = 8.9, df = 2977, p-value < 2.2e-16; ID: t = 9.8, df = 3001, p-value < 2.2e-16; CO: t = 8.3, df = 997, p-value = 2.2e-16; df: degrees of freedom). Conversely, for the highly experienced curators from MINT and BioGrid, this correlation was much weaker (BG: t = 1.75, df = 248, p-value = 0.08; MA: t = 1.6, df = 46, p-value = 0.12; MB: t = 3.2, df = 45, p-value = 0.0026; MC: t = 0.7, df = 51, p-value = 0.48; MD: t = 0.17, df = 22, p-value = 0.86). This is likely due to the fact that these curators are far more adept at recognizing relevant keywords and passages in the MEDLINE abstracts, as can also be seen by the very strong agreement on labels between the two databases (overlap 96%, Cohen's Kappa = 0.85).

Agreement with the expert curators was lower, as expected, but within acceptable ranges (for MINT vs. expert, 92% overlap, Kappa = 0.69; for BioGrid vs. expert, 91%, Kappa = 0.69). There was an overall agreement on labels (true, false) between all three groups (MINT, BioGrid, and expert) of 85.5% of all abstracts. This overlap should be compared to the highest accuracy (TP + TN/number of all articles) measured in the ACT, 89%. Furthermore, BioGrid and MINT follow (similar) in-house protocols for labeling the abstracts, while the expert annotations were done using a special protocol designed just for the challenge. This fact is likely to explain the better agreement between database curators than between the expert annotators and the curators.

#### ACT team results

In total, ten teams participated in this task. The individual results of each run are shown in Table 4, the team ID associations are show in table 3. For each of these participation methods, teams could submit five runs for a total of ten if they participated both offline and online. The highest AUC iP/R achieved by any run was 68%, the best MCC score measured was 0.55. The iP/R curve of the best team (73, S. Kim and W. J. Wilbur) in the ACT task is available in the supplementary material section (additional file 2). By using the BioCreative Meta-Server (BCMS) framework for participating online, we were able to measure the time it took the systems to report a classification.

**Table 4:** ACT participant results

Team	Run/Srvr	Accuracy	Specificity	Sensitivity	F-Score	MCC	AUC iP/R	Time_half
*TC*	*RUN*_*1*	*89.03*	*93.87*	*61.98*	*63.16*	*0.56733*	*68.98*	*30.13*

T65	RUN_1	88.68	97.64	38.57	50.83	0.48297	63.85	40.19

T65	RUN_2	87.93	93.07	59.23	59.82	0.52727	63.89	40.19

T65	RUN_3	67.05	64.19	83.08	43.34	0.34244	41.74	55.95

T65	RUN_4	73.68	74.13	71.21	45.08	0.34650	41.74	55.95

T65	RUN_5	88.00	94.40	52.20	56.89	0.50255	62.39	40.83

T70	RUN_1	56.45	49.70	94.18	39.62	0.31789	56.76	42.12

T70	RUN_2	87.41	96.11	38.79	48.32	0.43346	56.76	42.13

T70	RUN_3	81.92	83.61	72.53	54.91	0.46563	56.76	42.12

T70	RUN_4	47.77	39.04	**96.59**	35.95	0.27060	56.76	42.12

T70	RUN_5	86.84	98.62	20.99	32.62	0.34488	56.76	42.13

T73	RUN_1	87.55	91.81	63.74	60.83	0.53524	65.91	38.33

T73	RUN_2	**89.15**	94.95	56.70	61.32	**0.55306**	67.96	**37.10**

T73	RUN_3	87.78	92.61	60.77	60.14	0.52932	65.89	38.19

T73	RUN_4	88.88	94.34	58.35	**61.42**	0.55054	**67.98**	37.15

T73	RUN_5	87.62	92.18	62.09	60.33	0.53031	65.37	38.40

T81	RUN_1	59.03	58.76	60.55	30.96	0.13949	19.93	82.27

T81	RUN_2	58.47	57.86	61.87	31.12	0.14219	19.69	82.76

T81	RUN_3	25.37	14.72	84.95	25.66	-0.00344	15.66	102.73

T81	RUN_4	63.45	69.16	31.54	20.74	0.00538	16.20	104.95

T81	RUN_5	69.17	77.35	23.41	18.72	0.00645	15.63	98.72

T81	SRVR_9	84.88	**99.98**	0.44	0.88	0.05220	44.19	50.11

T81	SRVR_10	85.38	99.61	5.82	10.78	0.17771	50.25	45.11

T81	SRVR_11	84.73	99.86	0.11	0.22	-0.00272	46.02	48.23

T81	SRVR_12	84.30	98.86	2.86	5.23	0.05244	32.11	56.89

T81	SRVR_13	84.88	99.92	0.77	1.52	0.05791	18.59	113.11

T88	RUN_1	42.63	35.11	84.73	30.94	0.15238	21.97	84.90

T88	RUN_2	56.92	53.73	74.73	34.47	0.20417	26.04	75.33

T89	RUN_1	80.02	80.90	75.06	53.26	0.44911	61.29	41.31

T89	RUN_2	81.00	81.75	76.81	55.08	0.47242	62.13	40.99

T89	RUN_3	82.40	83.85	74.29	56.15	0.48180	60.48	41.72

T89	RUN_4	87.73	94.79	48.24	54.40	0.47967	43.76	43.09

T89	RUN_5	87.27	91.81	61.87	59.58	0.52082	48.47	44.57

T89	SRVR_4	77.80	77.84	77.58	51.46	0.43152	57.44	44.63

T89	SRVR_5	78.05	78.15	77.47	51.71	0.43424	57.56	45.20

T89	SRVR_6	79.90	81.00	73.74	52.67	0.44073	54.97	45.93

T89	SRVR_7	86.25	92.06	53.74	54.24	0.46156	41.58	45.94

T89	SRVR_8	86.87	90.39	67.14	60.80	0.53336	47.40	45.55

T90	RUN_1	88.73	95.15	52.86	58.73	0.52736	51.14	39.02

T90	RUN_2	88.70	94.97	53.63	59.01	0.52890	51.65	39.14

T90	RUN_3	88.32	93.93	56.92	59.64	0.52914	65.24	39.29

T90	RUN_4	88.93	96.03	49.23	57.44	0.52237	49.26	70.68

T90	RUN_5	88.60	95.05	52.53	58.29	0.52204	50.83	39.27

T92	RUN_1	86.22	90.77	60.77	57.22	0.49155	50.99	42.40

T100	RUN_1	88.77	96.82	43.74	54.15	0.50005	61.62	42.57

T100	RUN_2	88.27	93.89	56.81	59.49	0.52732	61.86	39.05

T100	RUN_3	81.13	82.69	72.42	53.80	0.45256	60.25	41.60

T100	RUN_4	81.85	82.85	76.26	56.04	0.48270	63.75	38.41

T104	RUN_1	80.12	80.69	76.92	53.99	0.45999	53.67	48.21

T104	RUN_2	80.07	80.47	77.80	54.21	0.46370	53.67	48.21

T104	RUN_3	64.93	59.86	93.3049	44.66	0.38161	53.67	48.21

T104	RUN_4	69.78	66.25	89.56	47.34	0.40530	53.67	48.21

T104	RUN_5	86.27	98.47	18.02	28.47	0.30064	53.67	142.95

Evaluation results based on the unrefined Gold Standard, in terms of Accuracy, MCC Score and AUC iP/R. The highest score for each evaluation column is show in bold typeface. Run/Srvr (RUN = offline run/SRVR = online run via the BCMS), MCC (Matthew's Correlation Coefficient), AUC iP/R (Area under the interpolated Precion/Recall curve). Time_half is the fraction of time needed to classify half of the positive abstracts using the output of that run when compared to unranked results. Note that some runs submitted by mistake the opposite ranking as requested for the negative records, which explains higher classification time (e.g. Team 104, RUN 5 with Time_half of 142.95), inverting in these cases the order of negative articles resulted in comparable time savings to the other systems.

A simple consensus prediction was generated using majority voting (see results in table 4), generating a ranking based on the percentage agreement derived from the different runs. This combined system returned the best MCC score (0.57), obtained a better AUC iP/R result than the best single run (68.98) and reached the best f-score (63.16).

The consensus prediction generates a ranking where the top of the list is enriched in positive articles while the end of the list has mostly negative articles. This means that the proportion of articles confirmed to be positive through manual curation following this ranking will be higher at the start of the process, which leads to a much greater manual curation yield when compared to unsorted revision.

**Figure 4:**
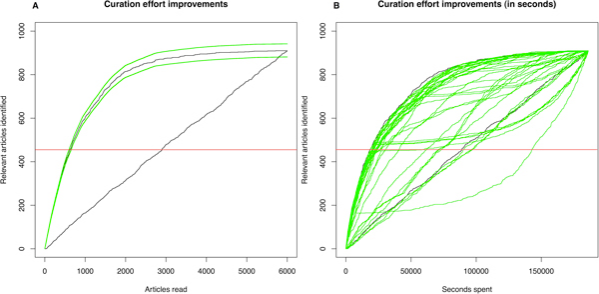
**ACT consensus analysis**. (**A**) The two black lines represent the number of relevant articles found while traversing the dataset. The diagonal line represents a random traversal, the parabolic line above represents a traversal following the ranking proposed by the consensus predictions. The green lines are bootstrap estimates for standard deviation. The horizontal red line represents half of the 910 articles. (**B**) Instead of showing the traversal in articles read, this shows the time spent reading them. The diagonal line represents a random traversal, the parabolic line above represents a traversal following the ranking proposed by the consensus predictions. The green lines represent traversals using the scores provided by the systems. Note that some runs seen below the random traversal seemed to have provided the opposite ranking for the negative documents than required, based on the submission format. TC: Team consensus prediction.

We have used the consensus predictions for the ACT task to illustrate this idea on Figure 4A. To classify half of the 910 relevant articles by examining randomly (i.e. unranked) records from the 6000 abstract test set would require reading 3000 abstracts, whereas when read in the order suggested by the consensus prediction, it would only require reading the top 7% (around 728, a reduction to 24.27% when compared to the 3000 articles that need to be read when not using ranking).

Consider T*_c_*(d) the time required to classify a document d into relevant or non-relevant. This time will in fact depend on many features, including the curator performing the classification, length of the document and the underlying annotation guidelines (see the discussion section for an analysis on curation times for different curation). In our case, the manual classification timings suggest that determining if an article is non-PPI relevant can be significantly faster than determining that it is relevant. This indicates that, although there is a great time saving obtained by using ranked system outputs, this is affected by both the proportion of relevant cases as well as the difference in classification time between relevant and non-relevant articles. Using the consensus system predictions would require only 30.13% of the time for finding half of the relevant documents in the test set collection when compared to random ranking of abstracts (no ordering/ranking). Figure 4B illustrates this idea, showing the number of articles against the time spent in classifying them (using the T*_c_*(d) from the real curators). This also shows the advantages over baseline selection based for instance on the use of a set of expert defined keywords for article selection, which moreover obviously does not generate a proper relevance ranking of the resulting hits. Using the presence a set of keywords provided by MINT curators as a way of selecting relevant articles resulted in a recall of 17.36%, a precision of 61.96% with F-score of 27.12%. Although the precision of this keyword-based selection is relatively acceptable, the overall performance is significantly lower than the one obtained by participating teams in general.

#### Participant technologies used for ACT

Participants were asked to fill in a short questionnaire after the test phase. Interestingly, four teams (73, 81, 92, 100) used other sources of training data than what was provided through the challenge itself (e.g., data from former BioCreative challenges or random likely-negative articles). We also asked teams to evaluate the difficulty of the task (easy, medium, hard); no team thought the ACT task was easy, four (73, 81, 100, 104) said it was hard, while the others classified it as 'medium'. All teams did some amount of lexical analysis of the text (sentence splitting, tokenization and/or lemmatization/stemming was done by all teams), and many included Part-of-Speech-tagging (POS) (teams 65, 73, 89, 90, 104) or even Named Entity Recognition (teams 65, 70, 73, 81, and 90). Teams 65 and 73 used dependency parsing on the abstracts. For generating their predictions all teams relied on the title and abstract, half used the MeSH terms, too, and one team was even also able to explore full text information for some of the articles. For feature selection or weighting purposes, approaches used by participating teams include statistical methods like Chi-Square, mutual information, frequency cut-off and Bayesian weights as well as other selection criteria such as the restriction to particular Part-of-Speech types. Teams 81, 89, 100 and 104 also used dimensionality reduction techniques on their features. A common characteristic of most of the participating teams was the use of machine learning techniques in general. Half of them used Support Vector Machines (SVM) for the classification (teams 81, 89, 90, 92, 100), and most of those combined the SVM with other supervised methods (81: (their own) Variable Trigonometric Threshold linear classifier, 89: Naïve Bayes, 90: Logistic Regression, 100: Nearest Neighbour). Team 70 used Nearest Neighbour, 104 Naïve Bayes, 73 Large Margin class with Huber loss function, and team 65 used a Maximum Entropy classifier. For ACT, Team 90 devised two independent systems one using SVM and the other using Logistic Regression.

### IMT

#### IMT annotation data

The annotations provided for the IMT task test set had been produced by the BioGRID and MINT database curators. Both follow a slightly different article selection criteria. In case of MINT, they carry out an exhaustive journal curation, examining each article for a specified time period given a selected journal, while BioGRID is primarily interested in curation of articles for a particular organism of interest. For this task, both databases agreed to use the same curation standards. Due to the considerable workload associated with producing the requested interaction detection method annotations for full text articles, the test set size was limited to 223 articles (see table 2). Nevertheless, for implementing their systems we were able to compile a large collection of training and development set articles with annotations, enough to build supervised learning methods that basically consider this task as a multi-class classification scenario. As only a small set of journals were used as test set, there were some differences in the distribution of methods across the three data collections provided, as shown in Figure 1.

#### IMT team results

In total, eight teams participated in this task. The official evaluation results of each run are shown in table 5, measuring the performance on the documents for which the system provided results (results averaged over the per-document scores are called "macro-averaged" results). The evaluation of the overall performance of the systems on the whole test set is shown in table 6 (i.e., calculated from the summed up true/false positive and true/false negative counts over *all *documents - "micro-averaged" results), while the corresponding team information details can be obtained from Table 3. As with the ACT, teams could participate offline, sending the results via e-mail, as well as online via the BioCreative Meta-Server (BCMS) [40]. In case of the macro-averaged results the highest AUC iP/R achieved by any submission was 52.974%, obtained by run 3 of team 90 (with an F-score of 54.616). Run 3 of team 70 reached the overall best precision of 80.00% at a recall of 41.50% (F-score of 51.508, but predictions are only available for 30 documents). Run 5 of Team 65 was the only one to achieve full recall (100%) while at the same time mantaining an AUC of 50.11%, just marginally lower than the best one. The most competitive F-score was of 55.06 (62.46% precision, 55.17% recall, based on 199 documents), obtained by Team 69 run 4. With respect to the micro-averaged results, the best prediction corresponded to run 3 of team 90. It obtained a precision of 52.30%, recall of 58.25%, F-score of 55.117, MCC of 0.5420, and AUC iP/R of 0.3542. By using the BCMS framework for participating online, we were able to measure the time it took the systems to report interaction method identifiers for full-text articles. However, there was only one team (89) participating online in this task, albeit with 5 servers and quite competitive results. This team annotated a full-text article on average in 3.7 seconds (sd: ±0.35 sec), and achieved a maximum F-Score score of 52% with an AUC iP/R of 48%.

**Table 5:** IMT macro-averaged participant results

Team	Run/Srvr	Docs	Precision	Recall	F-Score	AUC iP/R
*Base*_*Top4*	*RUN*_*1*	*222*	*28.60*	*47.75*	*33.78*	*0.32771*

*Base*_*Regex*	*RUN*_*1*	*153*	*33.88*	*22.31*	*24.73*	-

T65	RUN_1	**222**	9.35	83.21	16.32	0.47884

T65	RUN_2	**222**	2.45	**100.00**	4.75	0.44034

T65	RUN_3	**222**	9.99	79.38	17.16	0.47650

T65	RUN_4	**222**	33.48	42.88	35.40	0.30927

T65	RUN_5	**222**	2.44	**100.00**	4.74	0.50111

T69	RUN_1	214	54.87	57.91	52.39	0.52112

T69	RUN_2	211	57.01	57.35	53.42	0.51844

T69	RUN_3	203	60.24	56.41	54.45	0.51470

T69	RUN_4	199	62.46	55.17	**55.06**	0.51013

T69	RUN_5	190	64.24	52.44	54.35	0.49390

T70	RUN_1	143	51.78	35.01	37.84	0.31402

T70	RUN_2	72	71.76	36.81	45.61	0.36215

T70	RUN_3	30	**80.00**	41.50	51.51	0.41500

T70	RUN_4	205	31.65	38.72	31.75	0.32295

T70	RUN_5	159	36.36	21.26	24.75	0.18976

T81	RUN_1	**222**	4.44	63.91	8.19	0.22022

T81	RUN_2	221	9.39	41.92	14.12	0.19766

T81	RUN_3	**222**	13.51	28.35	17.41	0.17010

T81	RUN_4	**222**	13.21	29.57	17.34	0.20388

T81	RUN_5	209	21.93	24.64	21.34	0.18733

T88	RUN_1	219	29.10	45.04	33.60	0.38590

T88	RUN_2	220	28.67	45.53	33.35	0.38373

T89	RUN_1	200	54.78	53.37	50.91	0.46061

T89	RUN_2	200	54.95	53.23	50.76	0.46423

T89	RUN_3	201	54.05	53.25	50.23	0.45330

T89	RUN_4	199	54.48	54.18	51.25	0.47211

T89	RUN_5	201	55.30	56.12	52.38	0.47807

T89	SRVR_4	200	55.33	55.61	52.11	0.47636

T89	SRVR_5	199	54.09	54.00	50.96	0.47650

T89	SRVR_6	201	55.14	56.12	52.35	0.48047

T89	SRVR_7	203	50.46	55.66	50.06	0.47392

T89	SRVR_8	199	54.04	54.05	50.84	0.47534

T90	RUN_1	200	56.11	51.59	50.72	0.44687

T90	RUN_2	203	56.37	53.19	51.20	0.47159

T90	RUN_3	217	55.29	59.90	54.62	**0.52974**

T90	RUN_4	177	63.98	46.89	51.36	0.44118

T90	RUN_5	164	66.26	46.78	52.02	0.44458

T100	RUN_1	213	47.26	54.97	47.06	0.43312

T100	RUN_2	**222**	41.19	54.61	44.18	0.43238

T100	RUN_3	**222**	35.29	45.53	37.50	0.32459

T100	RUN_4	**222**	35.29	45.53	37.50	0.32459

T100	RUN_5	125	56.40	30.65	37.01	0.29387

Macro-averaged results when evaluating only documents for which the system reported results (i.e., measuring the average per-document performance only on the documents each run produced annotations for). The highest score for each evaluation column is show in bold typeface, the lowest in italics. Run/Srvr: RUN = offline run, SRVR = online server run via BCMS; Docs: number of documents annotated; AUC iP/R: Area under the interpolated precision/recall curve. Base Top4: baseline system that assigns the four most frequent classes ordered by their frequencies in the training/development set. Base Regex: simple matching strategy based on regular expressions.

**Table 6:** IMT micro-averaged participant results

Team	Run/Srvr	Precision	Recall	F-Score	MCC	AUC iP/R
*Base*_Top4	*RUN*_*1*	*28.60*	*48.20*	*35.90*	-	*0.15924*

*Base*_ *Regex*	*RUN*_*1*	*32.45*	*22.34*	*26.46*	-	-

T65	RUN_1	8.77	84.82	15.89	0.23552	0.27588

T65	RUN_2	2.45	**100.00**	4.78	0.06259	0.24484

T65	RUN_3	9.42	81.78	16.89	0.24172	0.27727

T65	RUN_4	33.48	42.32	37.39	0.36166	0.14169

T65	RUN_5	2.44	**100.00**	4.76	0.06193	0.29016

T69	RUN_1	52.07	55.03	53.51	0.52519	0.34302

T69	RUN_2	54.34	53.51	53.92	0.52958	0.33824

T69	RUN_3	57.36	50.29	53.59	0.52796	0.32539

T69	RUN_4	59.25	48.01	53.04	0.52456	0.31711

T69	RUN_5	61.33	43.64	51.00	0.50896	0.29373

T70	RUN_1	48.61	23.15	31.36	0.32617	0.12949

T70	RUN_2	70.00	11.95	20.42	0.28419	0.08731

T70	RUN_3	**80.65**	4.74	8.96	0.19270	0.03826

T70	RUN_4	31.22	36.43	33.63	0.32216	0.15688

T70	RUN_5	32.69	15.94	21.43	0.21717	0.05734

T81	RUN_1	4.54	66.03	8.50	0.11406	0.07716

T81	RUN_2	8.71	42.13	14.43	0.15560	0.06239

T81	RUN_3	13.51	28.46	18.33	0.17168	0.04657

T81	RUN_4	13.20	27.70	17.88	0.16667	0.05601

T81	RUN_5	21.35	22.20	21.77	0.20090	0.05283

T88	RUN_1	28.44	45.16	34.90	0.34146	0.20244

T88	RUN_2	28.17	45.92	34.92	0.34263	0.20069

T89	RUN_1	52.52	49.53	50.98	0.49997	0.28202

T89	RUN_2	52.02	48.96	50.44	0.49451	0.28589

T89	RUN_3	50.78	49.34	50.05	0.49016	0.27238

T89	RUN_4	52.50	49.91	51.17	0.50181	0.29220

T89	RUN_5	52.58	52.18	52.38	0.51382	0.29980

T89	SRVR_4	52.71	51.61	52.16	0.51163	0.29926

T89	SRVR_5	52.28	50.10	51.16	0.50168	0.30046

T89	SRVR_6	52.28	52.18	52.23	0.51226	0.30049

T89	SRVR_7	49.55	52.56	51.01	0.49972	0.29303

T89	SRVR_8	51.76	50.29	51.01	0.49999	0.29766

T90	RUN_1	53.33	47.06	50.00	0.49113	0.26805

T90	RUN_2	52.56	48.77	50.59	0.49625	0.28386

T90	RUN_3	52.30	58.25	**0.5512**	**0.54201**	**0.35423**

T90	RUN_4	61.09	38.14	46.96	0.47436	0.25209

T90	RUN_5	64.24	35.10	45.40	0.46707	0.24270

T100	RUN_1	44.59	51.61	47.85	0.46794	0.26055

T100	RUN_2	39.86	54.84	46.17	0.45448	0.26982

T100	RUN_3	35.29	44.59	39.40	0.38240	0.15734

T100	RUN_4	35.34	44.59	39.43	0.38271	0.15758

T100	RUN_5	54.86	18.22	27.35	0.30847	0.11109

Micro-averaged results when evaluating all documents (i.e., measuring the overall performance of each run on the whole document set). The highest score for each evaluation column is show in bold typeface, the lowest in italics. Run/Srvr: RUN = offline run, SRVR = online server run via BCMS; MCC: Matthew's Correlation Coefficient; AUC iP/R: Area under the interpolated precision/recall curve (micro- averaged by iterating over the precision/recall values of the highest ranked annotation of all articles, then all second ranked annotations, etc.). Base_Top4: baseline system that assigns the four most frequent classes ordered by their frequencies in the training/development set. Base_Regex: simple matching strategy based on regular expressions.

In order to interpret the performance scores it is important to put them into context. As already mentioned, some of the interaction methods appear more frequently in the training and development sets. This might lead to the assumption that such a distribution can be used to derive a sort of statistical baseline prediction, using the most frequent classes in the test/development set (brown colored classes in Figure 1) to establish a baseline qualifier. A simple statistical baseline was generated by assigning the four most frequent classes, constituting more than 50% of all assignments in the training/development set, to every test set articles, ranked/ordered by their frequency in the training/development set. The resulting scores are shown in tables 5 and 6. The improvement over this distribution baseline when considering submitted runs is +0.21283 (163%, from 0.33777 to 0.55060) in case of F-score and +0.20203 (162%) in case of AUC iP/R (from 0.32771 to 0.52974) from macro-averaged scores. As for micro-averaged scores, evaluated team runs improve the F-score by +0.1922 (154%, from 0.35901 to 0.55117) and AUC iP/R by +0.1950 (222%, from 0.15924 to 0.35423). However, the statistical baseline is not associated to any evidence text passages; The annotations cannot be interpreted by human curators, and thus have only limited practical value.

A very common approach to link lexical entries to free text is by using term-lookup strategies, often by using either string matching or more competitive matching strategies (e.g., regular expressions). To compare the value of the resulting tools to such a baseline regular expression method, an additional comparative analysis has been carried out. For the Regex baseline system, a regular expression is formed for each of the method names (concept name, not synonyms). The text is matched against each of the regular expressions, and if there is a successful match, the association to the corresponding method is reported. This baseline system was included in the distribution files on the BioCreative web page. The improvement of the best run over the Regex baseline in terms of macro-averaged f-score was +0.2128 and for the micro-averaged f-score even +0.2865. Aspects related to the method ranking (AUC iP/R) were not examined, as this system does not produce a proper ranking.

#### Participant technologies used for IMT

The participants were asked to fill in a short questionnaire, and all participants responded. Only one team (81) used other sources of training data than what was provided through the challenge itself, one team made use of the UMLS (69) and two of MeSH terms (90, 100). Most teams relied on the provided text we extracted using the UNIX tool 'pdftotext', while Team 100 made use of the PDFs directly. Most teams incorporated lexical analysis of the text (sentence splitting, tokenization and/or lemmatization/stemming), quite a few looked at n-gram tokens (teams 81, 89, 90, 100), but only one also included Part-of-Speech-tagging (team 90), and, interestingly, some teams omitted a specialized Named Entity Recognition approach (NER; teams 81, 89, 100; instead using regex matching). Team 90 even made use of shallow parsing techniques. All teams except 81 relied on Bag-of-Word vectors, and teams 70 and 88 did not use any supervised classifiers. Teams 90 and 69 were the only teams to use a Logistic Regression classifier trained on each term, team 90 also applied a Support Vector Machine, and team 69 used MALLET for NER. Other than that, no team reported use of existing BioNLP libraries, instead relying mainly on in-house tools. Only teams 90 and 65 applied gene/protein mention detection. In order to weight unigrams and bigrams features, team 89 calculated mutual information and chi square values. This team reported that these features were ranked the highest for them after feature selection, and that an additional feature for node popularity was very useful for this task. Chi square statistics were also used to score collocations and bigrams in case of team 65. We also asked teams to evaluate the difficulty of the task (easy, medium, hard); No team thought the task was easy, half (70, 89, 90, 100) said is was hard, while the other four (65, 69, 81, 88) classified it as 'medium'.

### Online participation

In addition to offline participation (sending the system results by email), the BioCreative Meta-Server (BCMS) [40] framework was provided to participants, using exactly the same setup as for BC II.5 [15]. This enabled online participation, submitting results via web service calls. This online submission allows measuring the time it takes systems to report results for the articles they annotate. However, there was only one team (89) participating online in the method sub-task (IMT) and only two (81 and 89) in the article classification sub-task (ACT). Team 89 annotated full-text articles (IMT) on average in a surprisingly short period of time - 3.7 seconds (sd: ±0.35 sec) - achieving a maximum F-Score score of 52% with an AUC iP/R of 48%. Team 81 annotated ACT Medline abstracts on average in 20 seconds (sd: ±12 sec) and although the maximum MCC score was only 0.11, their best AUC iP/R was 50% (Server 10). The second team, 89, did even better in the online ACT with an average of 1.9 seconds (sd: ±0.57 sec) per abstract, achieving a maximum MCC score of 0.61 (Server 8); their best AUC iP/R score was 58% (Server 5). The timing results match fairly well with the results found during BC II.5, although the combined time and performance of team 89 is an slight advancement over BC II.5 (best online ACT team in BC II.5, by MCC score: Team 9, Server 29, with MCC 0.583, average time 22 seconds/article). However, the results are not necessarily comparable, as the BC II.5 ACT was carried out on full-text articles, while for BC III Medline abstracts were used. Moreover, in BC II.5, a single journal with a different class imbalance was used as opposed to the range of curation relevant journals utilized in BC III. On the other hand, timing of annotating full-text IMT articles compared to the interacting protein normalization task (INT) of BC II.5 - also on full-text - can be seen as comparable on the basis of article sizes *if one disregards the fact *that the namespace of proteins (INT, II.5) is probably much larger than that of method names (IMT, III). The fastest server in BC II.5 (T42, S20), performed at 14 sec/article - compared to 3.7 sec/article by T89 (see above), a nearly 4-fold improvement.

### Individual system descriptions

All participating teams were requested to provide a short technical summary on the strategy used for participation in the PPI task. Team summaries are ordered based on the team identifier.

#### Team 65: Gerold Schneider and Fabio Rinaldi (ACT, IMT)

Team 65 (OntoGene) included the following members: Fabio Rinaldi, Gerold Schneider, Simon Clematide, Martin Romacker, Therese Vachon. The OntoGene research group at the University of Zurich has developed competitive tools for the extraction of mentions of protein-protein interactions (PPI) from the scientific literature through participation in previous BioCreative editions [11,41]. While Team 65 had previously obtained good results in the task of extracting supporting information about the interactions, such as experimental methods [42], this team never considered before participation to the ACT task, which appears at first sight a pure document classification task where an NLP-rich approach would not be able to provide a significant contribution.

The participation to the ACT task of BioCreative III was motivated by the desire to dispel this negative assumption through enrichment of a traditional machine learning (ML) approach with features derived from their PPI pipeline. Three of the Team 65 runs apply Maximum Entropy optimization (using the MEGAM tool [43]). Features include lexical items, MeSH annotations, plus crucially a score delivered by their PPI detection pipeline. Two runs used only results of their protein-protein interaction detection pipeline (as developed for BC II.5), for comparison.

The feature weights used for the test set were drawn from the development set only. Including the balanced (but therefore biased) training set degraded the results on the development set. To keep training efficient and prevent over-training, Team 65 used frequency thresholding and feature selection to cut the set of features to 20,000. The submitted runs optimize for different evaluation metrics. The results proved to be competitive, reaching 3rd or 4th rank for each of the measures selected by the organizers.

It is interesting to notice that the best system used a similar approach, based on a dependency parser. However, that team made a richer use of the features delivered by the parser. These results prove that an NLP-based pipeline for PPI extraction definitely provides a positive contribution towards the solution of the ACT task.

For the PPI-IMT task, the OntoGene group developed two statistical systems (called system A and system B here). Both are based on a Naïve Bayes approach but use different optimizations and heuristics. System B is a very generic Naïve Bayes multiclass classifier, whereas system A was optimized for IMT, taking into account terminological information obtained from the PSI-MI ontology.

For each of the two systems, two runs aiming at maximizing AUC and F-score were submitted. Additionally, a fifth run combining the max AUC runs of both systems was submitted. Best precision can always be obtained by taking only the best ranked method for each article, so no specific run aiming at optimizing precision was submitted.

Nearly all of the runs have very high recall, two of them even reaching 100% (no other participant system could reach full recall, the next best result was 66.03%). Run 5 is particularly remarkable as it combines full recall with high AUC iP/R (0.501), which is only marginally less than the best results in the competition - 0.5297, which however has much lower recall. For a semi-automated curation application, the configuration of near-total recall with good ranking is probably optimal. More details of this approach can be found in *Schneider et al*, same volume.

#### Team 69: Robert Leaman and Graciela Gonzalez (IMT)

Team 69 included the following members: Robert Leaman, Ryan Sullivan, Graciela Gonzalez. The system of team 69 modeled the detection of interaction methods in a document as a set of document-level classification problems. For each interaction detection method Team 69 trained one machine learning classifier to detect whether the method was mentioned at least once in an entire document. Each interaction detection method was classified independently, without regard for any subtypes or supertypes of the method. System input consisted of the *pdftotext *version of each article, and system output consisted primarily of the probability that each interaction detection method was mentioned somewhere in the document according to the classifier for that interaction method. This system also found support statements by applying the classifiers trained at the document level to each sentence in the document, then taking the sentence from the document with the highest probability output by the classifier as the support for the corresponding interaction detection method.

Preprocessing steps performed by this system include sentence-breaking, tokenization, normalizing case and Unicode characters, stop word removal [44] and stemming [45]. All classifiers used the same feature set, consisting of term and lexicon membership features. Term features are binary-valued and indicate the presence or absence of a single stemmed token within the document. Strict lexicon membership features are also binary-valued and indicate whether there was a sentence within the document that contained all of the tokens from any of the names of the detection method being located. Fuzzy lexicon membership features are similar to strict lexicon membership except that they are real-valued, representing the proportion of the tokens of the interaction detection method name that the sentence contains. Their lexicon of interaction detection method names was compiled primarily from the name, synonyms and unique identifiers (e.g. 'MI:0006') from the PSI-MI ontology [46]. Team 69 added approximately 40 additional synonyms by locating concepts in the UMLS Metathesaurus [47] from semantic types such as '*Laboratory Procedure*' which share a name with a concept in the PSI-MI ontology. All names in the lexicon were preprocessed in the same manner as the document text.

Logistic regression, as implemented by MALLET [48], was used for all classifiers. Documents from the training data annotated with a given interaction detection method were taken as positive instances of that method, and all other documents were considered negative instances of the method. No data was used for training other than the data provided for the task. The classifiers were trained using L_1 _regularization, via the orthant-wise limited memory quasi-Newton algorithm (OWL-QT) [49]. L_1 _regularization typically creates a sparse model, meaning that the weight of most parameters is set to zero. This is in contrast to L_2 _regularization, which usually learns many weights that approach zero asymptotically. L_1 _regularization therefore has many of the same advantages as feature selection, such as increased interpretability and allowing faster inference. In addition, L_1 _regularized models have been shown to be more robust to irrelevant features than L_2 _regularized models, since the amount of training data needed rises only logarithmically in the number of irrelevant features present [50]. Team 69 found in their experiments that training with L_1 _regularization resulted in approximately 3.3% higher F-measure and 4.9% AUC iP/R than training with L_2 _regularization.

#### Team 70: Sérgio Matos (ACT, IMT)

Team 70 included the following members: Sérgio Matos, David Campos, José L. Olivera. The proposed method of Team 70 for the ACT subtask makes use of the domain terminology in a vector-space classification approach [51]. Basically, the documents in the training set are represented as vectors of biologically relevant words, to which the unclassified documents are compared. The underlying lexicon includes a list of interaction methods from the Interaction Method Ontology (PSI-MI) [46], distributed by the organizers for the PPI-IMT task, and biologically relevant words, extracted from the BioLexicon resource [52]. Document vectors are stored as a Lucene [53] index with the following structure: the document identifier, for referencing purposes, the document classification (1 for relevant; 0 for non-relevant), and two text fields, one for the textual occurrences of the lexicon terms and the other for the corresponding lemmas. The use of lemmas allows normalizing related terms to a single lexical entry. In this case, the BioLexicon terms are normalized to the infinitive form of the verb (for example, 'interacts', 'interacting' and 'interaction' are all normalized to 'interact').

During the classification of a new document, each occurrence of a lexicon term (or the corresponding lemma) is added to the query string, which is then used to search the index. From this search, the top M documents are retrieved, together with the corresponding classifications and Lucene similarity scores. The class probability for the new document is then calculated as the sum of the similarity scores for each class, normalized by the sum of the scores for the M documents. A threshold, corresponding to the operating point of the classifier, is then used by Team 70 to select the class for that document. Term normalization, by using lemmas, allowed improvements in AUC iP/R between 3% (for M = 50) and 6% (for M = 500), compared to the use of the textual occurrence of the lexicon terms.

For the IMT subtask the approach followed by Team 70 was to find mentions of methods names and synonyms in the texts and apply a very simple heuristic to validate and rank the classifications. To facilitate approximate string searches, all documents in the test set were added to a Lucene index. This index is then searched for each entry in the dictionary of methods names - provided by the task organizers - and the top 100 documents for each search are retrieved. For synonyms of the same method (same PSI-MI identifier), the document scores are added together. Finally, a method ID is assigned to a document if that document/method score is above a preset threshold.

The use of domain terminologies and vector-space models for classification of PPI relevant documents provided encouraging results. The use of other lexical and ontological resources (e.g. Gene Ontology terms) may help improve the results obtained. Comparing to the use of classification models, such as SVMs, the proposed approach has the advantage that adding more classified documents as new information to the classifier only involves adding those documents, with the corresponding classification, to the index.

#### Team 73: Sun Kim and W. John Wilbur (ACT)

Team 73 included the following members: Sun Kim, W. John Wilbur. Protein-protein interactions (PPIs) in biomedical literature can be interpreted as a series of dependency relationships between words. Hence, capturing this information is key in detecting PPI information at both the article and sentence level. The main focus of Team 73 in the ACT task was to explore the usefulness of the syntactic information in addition to conventional approaches. The system proposed by Team 73 has three different modules, gene mention detection, feature extraction, and classifiers. The feature extraction module consists of two parts, word-based and relation-based feature extraction. Word-based features include the common feature sets such as n-grams and strings. Relation-based features are basically a set of dependency relationships between words at the sentence level.

As the first step of the filtering process, gene and protein names are tagged using the Priority model [54]. This step is essential because, in PPI events, protein names are the most important words triggering PPI descriptions. The Priority model was developed in the group of Team 73 to overcome the pitfall of other statistical approaches by emphasizing the right-most words in a candidate phrase. Next, gene-tagged articles are further processed to obtain features for a data-driven classifier. The highlighted feature in word-based extraction is MeSH terms. MeSH is a thesaurus for indexing and searching biomedical literature, hence this controlled vocabulary is a good indicator of an article's topic. Relation-based features investigate the dependency relationships between words. By using a dependency parser [55], a head word and a dependent word are determined as a two-word combination. Furthermore, gene names are alternatively anonymized by replacing a specific gene name with a common tag, e.g., 'PROTEIN', which reduces the total number of features while leaving dependency information intact. Another aspect of features considered by Team 73 is to extract higher-order patterns by evaluating a set of feature combinations. This process adds combination features that are detected as useful for the classifier. The last step of PPI article filtering is to learn to classify articles based on the extracted features. The constraint here is to minimize computational cost and processing time with reasonable classification performance. To achieve this purpose, a large margin classifier with Huber loss function [56] was adopted by Team 73. The Huber classifier is a linear predictor using a simple gradient descent learning algorithm, which results in excellent performance competitive with support vector machine classifiers.

Although the current approach has room for improvement, it produced the top-ranked performance in the BioCreative III ACT task by achieving 89.15% accuracy, 0.55306 MCC, 61.42% F-score, and 67.98% AUC in different data/feature combinations. As a result, Team 73 found that syntactic patterns along with word features can effectively help distinguish between PPI and non-PPI articles, in particular, with a limited training corpus. More details of this approach can be found in [57].

#### Team 81: Luis Rocha (ACT, IMT)

Team 81 included the following members: Luis M. Rocha, Anália Lourenço, Michael Conover, Azadeh Nematzadeh, Fengxia Pan, Andrew Wong, Hagit Shatkay. For the ACT Team 81 participated in both the online submission via the BioCreative MetaServer platform, as well as the offline component of the Challenge. Team 81 used three distinct classifiers: (1) the previously developed Variable Trigonometric Threshold (VTT) linear classifier [58,59] which employs word-pair textual features and protein counts extracted using the ABNER tool [60], (2) a novel version of VTT that includes various NER features as well as various sources of textual features [61], and (3) a Support Vector Machine that takes as features various entity count features from the NER tools team 81 tested. In addition to testing the power of available NER tools for classification of documents relevant for Protein-Protein Interaction, members of Team 81 were interested in investigating the advantages of using full-text data on the classification. Team 81 utilized the following NER tools and dictionaries: ABNER, NLProt, Oscar 3, ChEBI (Chemical names), PSI-MI, MeSH terms, and BRENDA enzyme names. Team 81 also used the output of their Interaction Methods Task pipeline as an additional annotation tool. While their submitted results suffered from many errors due to NER pipeline integration, Team 81 has since fixed the errors and obtained excellent classification results on training data, even when using simply feature counts (e.g. number of methods identified by PSI-MI in a document) from a few NER tools [61]. To address the IMT, Team 81 employed a statistical approach. Unlike most teams, which used NLP and/or classification of documents into the many different possible classes - corresponding to the many candidate methods - team 81 looked directly within the text for experimental evidence. That is, Team 81 looked within the text for candidate short passages likely to indicate experimental methods, used simple pattern matching to identify the method within the passage, and ranked candidate matches according to statistical considerations. To find candidate passages in text, Team 81 used classifiers they have developed independently [62], which were trained on a corpus of 10,000 sentences from full-text biomedical articles, tagged along five dimensions: focus (methodological, scientific or generic), type of evidence (experimental, reference, and a few other types), level of confidence (from 0 - no confidence, to 3 - absolute certainty), polarity (affirmative or negative statement), and direction (e.g. up-regulation vs. down-regulation). This corpus is available at: http://www.ncbi.nlm.nih.gov/pmc/articles/PMC2678295/bin/pcbi.1000391.s002.zip. While this corpus was not concerned with protein-protein interactions, the classifier trained on the Focus dimension showed high sensitivity and specificity in identifying Methods sentences. Using the text files provided by the BioCreative organizers, Team 81 broke the corpus into sentences (modifying the Lingua-EN-Sentence Perl module [63]), and eliminated bibliographic references using simple rules. The remaining sentences were represented as term vectors and classified according to their Focus, utilizing an SVM classifier [62], thus identifying candidate sentences that may discuss methods. Method Identifiers were then associated with the latter sentences by simple pattern-matching to PSI-MI ontology terms. The matches were then scored using a strategy described later in this volume, and high-scoring methods were reported along with the sentences as evidence.

#### Team 88: Ashish V Tendulkar (ACT, IMT)

Team 88 included the following members: Ashish V Tendulkar, Aniket Rangrej, Vishal Raut. Team 88 developed a maximum entropy classifier for PPI abstract classification task. The goal was to develop a classifier that takes biologically relevant clues as features while classifying the abstract. Team 88 used PSI-MI ontology for extracting features from the abstract and the title. They developed a dictionary based tagger for tagging mentions of PSI-MI concepts from different levels. In addition to these features, Team 88 used MESH terms provided along with the abstract. On training data, Team 88 obtained 35% precision and 55% recall. They believe that this is an interesting direction of classifying abstracts by constructing biologically relevant features. The ACT classification system will be available at the following URL: http://www.cse.iitm.ac.in/~ashish/research/ACT/

Team 88 developed dictionary based NER for detection of interaction method mentions from text converted PDF files as provided by the organizers. The system has the following components: (i) Dictionary construction; (ii) Pre-processing of scientific article, which includes sentence splitting and division into various sections; (iii) Interaction method NER; (iv) Post-processing. The dictionary was constructed by extending PSI-MI ontology to incorporate common variations of interaction methods used in scientific literature. The common variations include lower case lexicons, replacing space with hyphen, etc. The analysis of training data revealed that the interaction methods are definitely mentioned in the experimental method section and at times in the abstract and title of the research papers. Team 88 further observed that the extraction from title and abstract gives the best precision partly due to their original availability in text form and hence does not contain noisy characters as introduced in other parts due to PDF to text conversion. Thus, detecting these mentions reduces the number of false positives that would have been obtained from other sections of the paper. Further, Team 88 also observed that detecting all mentions in experimental methods section is sufficient to ensure high recall of the system. In order to take advantage of these observations, Team 88 first divided the article into different parts along the section boundaries. The standard division included abstract, introduction, results, discussion and experimental method. The main challenge here is to detect section headings from the article in text format obtained via PDF to text (*pdftotext*) conversion. Team 88 used several clues for detecting them: (i) The section header is usually in upper case or capitalized; (ii) they have standard names, which vary from journal to journal. Ideally, Team 88 would like to detect these names independent of journals, but in this implementation, Team 88 relied on a manually constructed dictionary of journal-specific section headers. They first obtain the journal in which the paper has appeared and use the appropriate section headers. Team 88 ignored the list of references and other sections like acknowledgements, but notes that the acknowledgement section contains strong clues about interaction detection method, since it contains references to certain facilities used for conducting the protein-protein interaction detection experiments. Team 88, however, has not exploited these clues in their current implementation. Team 88 then divided each article into sentences and each sentence is assigned a tag denoting the section in which it is appearing. Team 88 implemented a dictionary based NER system for detecting interaction method mentions. They first detected the mentions from the section describing experimental procedure, then title and abstract, and then from the remaining sections. The mentions from abstract and experimental procedure section were assigned highest confidence score. During the post-processing step, Team 88 disambiguated between the interaction methods with overlapping tokens. They chose the interaction method with longest match and discarded the mention subsumed in the larger entity. The IMT extraction system will be made available at the following URL: http://www.cse.iitm.ac.in/~ashish/research/IMT/

#### Team 89: Shashank Agarwal and Feifan Liu (ACT, IMT)

Team 89 included the following members: Shashank Agarwal, Feifan Liu, Hong Yu. Team 89 participated in both PPI tasks. For the article classification task (ACT), supervised machine learning algorithms Support Vector Machines (SVMs) and multinomial Naïve Bayes (NB) algorithms were trained on the training data. Unigrams (individual words) and bigrams (two consecutive words) were used as features for the classifier. The mutual information between each feature and the class label was used to rank those features and either the top 400 or top 1000 features were selected. For training, the training corpus of 2280 articles was combined with the development corpus of the 4000 articles. As the distribution of articles in the development corpus was the same as the distribution expected in the test data, for some runs, only the development corpus was used for training. The classifiers were trained using the freely-available Simple Classifier program https://sourceforge.net/projects/simpleclassify/ that was also developed by Team 89, which is based on the Weka1 framework.

The interaction methods task (IMT) was also approached as a binary classification task. Each node in the PSI-MI sub-ontology was considered to be independent of other nodes in the ontology, and an article-node pair was considered as positive if the corresponding interaction method was detected in that article, and negative otherwise. The nodes were preprocessed; for some nodes, synonyms were added manually, for example, 'anti bait immunoprecipitation' for 'anti bait coimmunoprecipitation'. The concept name of a node and all synonyms of the node were normalized (lowercased and lemmatized) and used separately. From each node's concept name and synonym names, unigrams and bigrams were extracted. For each unigram and bigram, the mutual information score and chi-square score were calculated. Machine learning classifiers were trained for IMT using 21 features. The features included checking if the node's concept or synonym name appears in the article, checking if the node's concept or synonym names' unigrams or bigrams appear in the article, and the sum of the mutual information score and chi-square score of the unigrams and bigrams that appear in the article. The frequency with which a node appears in the training data was also used as a feature. Using these features, the OntoNorm framework https://sourceforge.net/projects/ontonorm/ was developed for this task, where machine learning algorithms Random Forest, Random Committee, Naïve Bayes Tree and J48 were explored. OntoNorm is based on the Weka [64] framework as well.

#### Team 90: Xinglong Wang and Rafal Rak (ACT, IMT)

Team 90 included the following members: Xinglong Wang, Rafal Rak, Angelo Restificar, Chikashi Nobata, C.J. Rupp, Riza Theresa B. Batista-Navarro, Raheel Nawaz, Sophia Ananiadou. Pre-processing: Both IMT and ACT documents in plain-text format were pre-processed using a number of linguistic processors, including tokenisation, lemmatisation, part-of-speech tagging and chunking. The documents enriched by the linguistic features were then processed with a named entity recogniser, the same as used in our semantic search engine Kleio [65], which exploits dictionaries and machine learning methods to tag the following types of entities: genes, proteins, metabolites, organs, drugs, bacteria, diseases, symptoms, diagnostic/therapeutic procedures and phenomena.

Additionally, for each IMT and ACT document, Team 90 retrieved its MeSH headings. The information of interest included descriptor names and identifiers, in both their atomic and hierarchical form, with the latter more closely representing the underlying structure of MeSH. For IMT, Team 90 also manually constructed a mapping from the 10 most frequent MI IDs (Molecular Interaction Ontology concept identifiers) as found in the training data to their corresponding MeSH descriptors.

IMT: Team 90 approached IMT from two different angles. One was based on a commonly used multi-class, multi-label document classification framework. The other one involved translating the multi-class, multi-label classification to a binary classification problem by classifying an exhaustive set of pairs of PSI-MI synonyms and text phrases (chunks) found in the documents. Team 90 experimented with Logistic Regression (LR) and SVMs as underlying machine-learning methods for the former and SVM only for the latter, which we refer to hereafter as m-LR, m-SVM, and b-SVM, respectively. For m-LR and m-SVM they trained a series of binary classifiers, each corresponding to a single interaction method, using the one-vs-all strategy. For each model the positive instances constitute the documents that are assigned the interaction method for which the model is being built. The feature set used in training included the type and text of named entities, words surrounding the entities and the title of the section in which the entities occurred, as well as information about whether word unigrams and character n-grams from the MI definition and synonyms match those in the surrounding context of the entities.

During classification, a document is scored by all the models and the decision whether it should be assigned an interaction method is based on a thresholding strategy. Team 90 primarily experimented with two thresholding strategies, the local (class-specific) score-based optimisation strategy, as well as its more commonly used variant, the global (one for all classes) strategy. Both strategies assign a class to a document based purely on the score between the two and a given (local or global) threshold. Team 90 observed that the performance of their systems was improved considerably using the thresholding strategies when compared to applying the nominal threshold (probability equal to 0.5 in the case of LR, and distance to a hyperplane equal to 0.0 in the case of SVM).

The b-SVM approach first explores noun phrases (NP) and verb phrases (VP) in a document and collects those that are approximately similar to an interaction method name in the PSI-MI ontology. The strength of similarity is determined by a string similarity measure. An instance - a pair chunk-method - is considered positive if the document the chunk is coming from is assigned the interaction method in the pair. Team 90 trained an SVM model by extracting a rich set of features from each pair in the training data.

The features included the string similarity score, the chunk's adjacent words and named entities, information about whether the named entities occur in the definition of the method, the title of the section where the text chunk occurs, and information whether the document's MeSH headings match the interaction method based on the manually created mapping. The analysis showed that the most valuable features were named entities.

Team 90 also created simple ensemble systems by taking combinations of unions and intersections of classification outcomes produced by the above-mentioned systems. As tested in a cross-validation setup, the three highest performing ensemble systems were the union of m-SVM and b-SVM, the intersection of m-LR and b-SVM, and the intersection of all the three systems.

ACT: To get a better understanding of the task at hand, Team 90 analysed a few randomly chosen positive and negative sample abstracts from the training dataset, in terms of whether the presence of certain attributes in an abstract correlates with the assigned class (either positive or negative). The attributes included protein names, verbs or nominalised verbs around protein names that signify protein interaction (involving more than one participant), verbs or nominalised verbs near protein names signifying protein modification (involving only one participant), as well as protein-related and biochemical-process-related MeSH headings.

The features used in machine learning included the bag of words, named entities, protein context words (in sentences that contain at least one protein) with position information, and MeSH headings associated with the document. Similarly to IMT, Team 90 adapted LR and SVM classifiers. The analysis showed that the most discriminative features were the bags of words followed closely by protein contextual features and MeSH headings, especially their hierarchical representation.

#### Team 92: Keith Noto and Charles Elkan (ACT)

Team 92 included the following members: Keith Noto, Charles Elkan. The submission for the ACT of team 92 was the output from a system named PMAC (PubMed Article Classifier) [66] that classifies and ranks biomedical articles based on features extracted from PubMed, and based only on *positive *training examples. Because the submission was the result of a fully automated system that needs no tuning of parameters, only one run was submitted. The PMAC system is currently available free for public use at http://www.cs.tufts.edu/~noto/pmac. It is based on a tool developed with NIH funding to help maintain and expand TCDB, the Transporter Classification Database http://www.tcdb.org[67].

The PMAC system has wide utility because in many article classification tasks, the only available labeled examples are positive. Biomedical databases typically do not provide examples of articles that are *not *representative of their domains of interest. PMAC uses a supervised classification algorithm to distinguish between positive and unlabeled articles. The system then adjusts the trained model mathematically to account for the fact that a small fraction of unlabeled articles are actually positive; for details see Elkan and Noto, KDD 2008 [68]. The features that PMAC extracts from PubMed are

• Words in an article's abstract,

• Words in its title,

• Author names and affiliations,

• Journal name and publication type,

• Chemical substances mentioned in the paper, and

• MeSH descriptor and qualifier names.

Users may select some of these feature groups to exclude if desired, and they may select a subset of journals from which to present articles. For each article, the output of the classifier is the probability that the article is relevant. For classification, one may choose a threshold probability. Or, if the class imbalance is known, PMAC returns the appropriate percentage of test set articles (this is what Team 92 did for BioCreative III, ACT). Currently, PMAC uses an SVM classifier with a linear kernel function, strength of regularization selected using cross-validation on training data, and Platt scaling to estimate probabilities if necessary. Feature weighting and feature selection are not used. However, PMAC is compatible with any supervised classifier that is capable of ranking articles. The classification and ranking time of PMAC is customizable to a degree, because it depends on the size of the training set and in particular on how many unlabeled articles are included; the user may choose to limit this number. In the BioCreative III article classification task, Team 92 used approximately 10,000 unlabeled articles and the classification and ranking was done in about 17 minutes (not including article download and feature extraction time, which may take about 1-2 seconds per article, but need only be executed once per article, and can be done separately beforehand). The process of retraining and reclassifying articles can take place periodically (*e.g*., overnight), so that PMAC users can look for relevant articles instantly as needed. Team 92 discusses the running time further and show the superior accuracy of this approach, in terms of precision and recall, compared to hand-crafted rules in Sehgal *et al.*, 2011 [69].

The PMAC system automatically extracts article features from PubMed, so the only input needed is a set of PubMed ID numbers. Users of PMAC do not need to provide any features, nor do they need to understand the features used by PMAC. Compared to other submissions, there were two significant differences in the way Team 92 used the training data provided for the Biocreative III article classification task. First, they did not use the provided negative training instances (although they did note the class imbalance). Second, they ignored all the given training features, and used only features extracted from PubMed by PMAC.

These differences presumably put the PMAC system at a disadvantage, since it ignored a large amount of relevant information. However, PMAC performed better than the majority of participating systems, and achieved F-score within a few percent of the F-score of the best submitted run. Therefore Team 92 can recommend with confidence that biomedical researchers looking for an easy-to-use solution for classifying and ranking articles should try PMAC.

#### Team 100: Zhiyong Lu and Rezarta Islamaj Doğan (ACT, IMT)

Team 100 included the following members: Zhiyong Lu, Rezarta Islamaj Doğan, Aurelie Neveol, Minlie Huang, Yi Yang. This team used machine learning for predicting whether or not an article is about PPI in the ACT task. In addition to the training and development data sets of BioCreative III, Team 100 used similar data sets from BioCreative II and II.5. A noticeable aspect of their work is that in response to the class imbalance issue (ratio of positive/negative instances was roughly 1 to 5 in the BioCreative III development set), they recruited additional negative instances by including MEDLINE articles that are similar to the existing negatives using PubMed related articles [70,71]. Their principle learner for machine learning is an SVM like classifier that uses modified Huber loss function for handling large-scale data [56]. In terms of features Team 100 experimented with various kinds, ranging from words to neighborhood documents. They first learned separate models for each feature type and then merged the results of different features when making final predictions. Team 100 submitted a total of four offline runs. They investigated three types of word-based features with or without feature selection. In addition to the traditional bag-of-words and bigrams (two consecutive words), this team extended bigrams to any two co-occurring words in the same sentence (co-occurring words). The 4th feature type is a set of character strings of length 8 generated by shifting an 8-character window from the beginning of a sentence to the end. For all these feature types, stop words were retained. In particular, stop words were found useful in bigram features (e.g. interacting with). When feature selection was applied, Team 100 iteratively evaluated the importance of each individual feature through examination of their weights and subsequently removed 1,000 features with the lowest weights (i.e. closest to zero). In the 1st run, only the bag-of-words feature was used along with feature selection. In their 2nd and 3rd runs, the bigrams, co-occurring words and strings-of-length-8 features were used individually to yield three separate prediction results. In making the final prediction, a test article was predicted to be PPI relevant if and only if all three individual predicted classes were positive. Feature selection was used in the 3rd run, but not in the 2nd. In the 4th run, Team 100 combined 4 scores, two of which were produced by the machine learner using the bag-of-words and bigrams features. Two additional scores were computed: One is based on the similarity scores of a test document to its 10 closest neighbors. Specifically, the 10 scores were separated into two groups, indicating either a positive or negative relation to PPI among those neighboring documents. This team summed the scores of each group and used their difference as a score in the 4th run. The other score is based on the pattern matching method previously developed for detecting PPI methods (e.g. pull down) in the IMT task. Pattern matching was applied to an article's title and abstract and the number of PPI methods retrieved was used as a feature score. All four scores were fed to a log-linear model in producing a final prediction in the 4th run. Their results on the official test set show that they achieved the highest F-score of 59.49% and MCC of 0.527 in Run 2 while the best AUC iP/R of 63.75% was achived in Run 4.

Team 100 used two separate approaches (knowledge- and learning-based) for the IMT task. They designed and conducted their experiments mainly on the BioCreative III data. This group also used BioCreative II data and additional annotations from the MINT database, resulting in a total of 3,764 additional articles with PSI-MI codes. For all these articles, title and abstract text were extracted. Furthermore, when full text was freely available from PubMed Central, figure captions and text from material and methods sections were also extracted. In the knowledge-based approach, Team 100 investigated three individual methods and their performance on different text sections when applicable. The three methods are respectively based on directly matching PSI-MI terms and synonyms (Pattern Matching), on retrieving similar PSI-MI codes from neighboring documents (Nearest Neighbors), and on inferring PSI-MI codes from corresponding Medical Subject Headings (MeSH) indexing terms (MeSH to PSI-MI). Based on the results on the BioCreative III training data, Team 100 observed that prediction performance varied significantly from method to method for a given PSI-MI code. Therefore, their knowledge-based approach is code-specific: for each code Team 100 selected the best-performing method. For instance, the pattern matching method was selected for molecular sieving (MI:0071) while nearest neighbors method for two hybrid (MI:0018). Their novel learning-based approach formulates the prediction of PSI-MI task as a ranking problem such that the relevant codes should be ranked higher than those irrelevant ones. In the IMT task, for a target document Team 100 first obtained a pool of candidate codes from its similar neighboring documents. Next, for ranking, each code was represented by a vector of features, which ranged from word features (e.g. name feature indicating if a code name can be found in an article) to neighborhood features (e.g. how many neighboring documents are assigned a particular code). The ranking algorithm they applied is a listwise learning-to-rank algorithm named ListNet [71] because it naturally fits the problem in that each article typically contains a list of relevant codes (as opposed to one per document). Team 100 optimized the learning function by conducting cross validation experiments on the BioCreative III training and development sets. Finally, the ListNet algorithm produced a score for each candidate code and they empirically determined the top K ranked codes to be the answers of the target document. This team submitted five offline runs. The first run was based on the knowledge-based approach. In the second run, they combined the pattern matching and nearest neighbor methods by selecting PSI-MI codes from neighboring documents if the PSI-MI codes were also retrieved by pattern matching. The third and fourth runs were both based on the learning approach with the minor difference in K (K is always 3 for run 3; K varied depending on a score threshold in run 4). Run 5 of this group was optimized for precision by combining the results of Runs 2 and 3. Official results on the test set show that their first run yielded the best performance from all submissions of Team 100 (0.478 in F1-score when evaluated on the whole document set), indicating that it is somewhat helpful to choose methods based on individual PSI-MI codes.

#### Team 104: Jean-Fred Fontaine and Miguel A. Andrade-Navarro (ACT)

Team 104 included the following members: Jean-Fred Fontaine, Miguel A. Andrade-Navarro. Medline Ranker, implemented by Team 104, is a fast document retrieval tool that classifies the biomedical scientific bibliography in the MEDLINE database according to any selected topic. It applies a linear Naïve Bayesian classifier (LNBC) on scientific abstracts with a high processing speed (approximately 18000 abstracts per second) and a high precision [72]. The Medline Ranker web server http://cbdm.mdc-berlin.de/tools/medlineranker offers alternative query mechanisms through PubMed queries, MeSH terms or custom PubMed identifier (PMID) lists. In particular, it allows the selection of a training set, a background set, and a test set represented as PMIDs, which Team 104 used for the PPI abstracts classification task (ACT) of the BioCreative III challenge. To favour the speed and flexibility of the Medline Ranker system Team 104 focused its implementation on data pre processing and storage [72]. The complete XML version of MEDLINE is stored locally and weekly updated. English abstracts are stored in a MySQL database after part-of-speech processing used to define an abstract's profile as its set of nouns. A stop word list is used to remove common and non meaningful terms. Multiple occurrences of nouns in a single abstract are not taken into account [72,73]. A user's request for classification of a query set of abstracts requires the definition of a training set of abstracts and to choose a background set of abstracts. Upon a query, first, a LNBC is trained on the training set of abstracts by comparing their profiles to those of the background set of abstracts. Secondly, the abstracts in the query set are scored with the trained LNBC and P-values (based on a simulation on 10 000 randomly chosen abstracts) are associated to abstracts representing the confidence in classification. For the BioCreative III challenge, P-values were transformed in scores by subtracting the P-value to 1, after truncation of the P-value to ]0,1[. Medline Ranker uses for abstract classification only words from abstracts and therefore it does not depend on the quality or comprehensiveness of external data or annotations (i.e. MeSH or Gene Ontology terms). To produce fair results, Medline Ranker was used to process BioCreative III PPI ACT task data by training its algorithm and tuning the parameters only on the provided training set. Even if not as accurate as SVM classifiers, training a LNBC is significantly faster and it allows the tool to process millions of abstracts with comparable performance in a practical time [73]. For the PPI ACT task, the mean Medline Ranker run total duration was 1.29 seconds to process 8280 abstracts. Notably, the running time also depends on the MySQL search engine used for data access. Even if not specialised in the topic of PPI (e.g. using specific information extraction methods), the tool may be of interest in this task because it is freely available on the Internet and it can scan the ever growing scientific literature in a few minutes.

## Discussion

### ACT

Given the performance of systems, for example high-AUC-iP/R servers, it is likely that humans could make use of the results to quickly identify the most relevant articles in a set. Therefore, the time spent by the text mining pipelines should be put in contrast to the time a human would need to select relevant articles. This exact time will be established in future work with the annotators and curators who provided the Gold Standard. We have shown reasonable indications that online, automated systems could have a strong impact on reducing the time required to locate relevant articles. This aspect of quantifying automated versus manual classification effort constitutes a complementary approach to measures of performance explored in the popular TREC Genomics tracks, such as the mean average precision (MAP), which measures precision after each relevant document is retrieved for a given query [24].

As described in the data preparation section, during the manual classification of abstracts, the MyMiner system allowed the use of positive and negative keyword highlighting to improve text visualization. These terms were generated through inspection of instances during the training set construction and contained 374 negative and 73 positive terms. In order to determine whether they are actually present in the test set, we used all words in the test set collection, stemmed them, removed stopwords and generated unigrams and bigrams. Then the occurrences of each term (both unigrams and bigrams) were computed and their their frequencies using the Kullback-Leibler (KL) divergence were compared (smoothing 0 to 0.000001 and 1 to 0.999999). The corresponding formula is calculated by: *pos *· log(*pos/neg*) + *neg *· log(*neg/pos*); where *pos *and *neg *are the frequencies of documents containing the term in the negative and positive sets. When analyzing the 3,000 most significant terms resulting from this computation and comparing them to the set of terms used for manual highlighting, only 17.0% (361) of the manually defined negative terms were encountered in the top 3,000 terms as opposed to 61.5% (52) of positive terms. This illustrates that in general there is a greater textual diversity within the non-PPI relevant articles and that finding positive keywords and features that could be used for highlighting to improve manual inspection is of greater practical value.

Additionally, we examined articles that were difficult to classify correctly by automated systems. This analysis was based on the number of runs predicting incorrect labels on an article and shows how the agreement between automatic runs could actually be useful to detect wrong manual classifications. A total of 99 records had been predicted by more than 80% of the runs incorrectly, out of which 74 records corresponded to false negative records (labeled true by the annotators) and 26 corresponded to false positive cases (labeled false). Examining the latter showed that 16 of these 26 cases were incorrectly labelled by the annotators, and were in fact true PPI articles as indicated by the run agreements. Some of these wrongly labeled cases corresponded to abstracts describing oligomerization of DNA binding proteins and other cases where it was difficult to distinguish between an actual macromolecular structure (e.g. some channel) and an individual protein forming this structure. Also, cases of very specific subtypes of interactions (phosphorylation, acetylation and ubiquitination) were more difficult for manual labeling. In case the predictions were real false positives (i.e., correctly labeled), they could be assigned to some general example cases: (1) interactions between proteins and RNA, e.g. PMID:19447915, (2) between proteins and cellular structures (especially cell membrane, e.g. PMID:19690048), (3) complexes of proteins with inhibitor molecule compounds, e.g. PMID:19458048, (4) protein-DNA binding, e.g. PMID:19605346, (5) protein-compound binding (e.g. lipopolysaccharide), (6) mentions of components of a complex but without detailing PPIs, (7) genetic interactions and transcriptional activation, e.g. PMID:19596907, (8) interaction of a particular residue with some ion, (9) regulatory relations (phosphorylation dependent on certain protein) where it is not clear if an actual direct PPI is described and (10) relations between a pathway or signaling process and a phosphorylation event. Sometimes, several of these topics were found in a single abstract. In addition, among the false positives were abstracts dealing with descriptions of the spliceosome and general ubiquitination events. Interestingly, a number of these types of false positives were precisely the cases that had to be refined when preparing the annotation guidelines and had been specifically added as criteria for classifying abstracts as non-relevant for manual annotation during the initial rounds of annotation refinement. Looking at the records classified as false negatives (by at least 80% of the runs) it became clear that many of these records corresponded to abstracts discussing aspects related to host-pathogen interactions, inflammation and immune mechanisms. Unclear cases, even for humans, included associations of proteins with lipoproteins, certain types of breakdown or cleavage of proteins, descriptions of chimeric proteins and experimentally tagged fusion proteins. Commonly missed records included cases of a receptor protein binding to a ligand protein. This particular topic was also added during the annotation guideline refinement upon a request made by the expert curator, where a specific question was whether the mention of 'insulin receptor' corresponds to an implicit interaction of insulin with the insulin receptor. Among the false negative set are ambiguous cases that mention a heterodimer but where it was not very clear who the binding partner is, or records with very limited context information requiring domain expert knowledge to determine that a given protein pair mentioned is involved in a complex.

Overall participating teams used a variety of different features, many of them for training machine learning approaches. Among the explored features one can point out:

*1) Word token features*: unigrams (Bag-of-words), multi-word n-grams (mainly bigrams and trigrams), collocations/co-occurring words (word-to-word relationships).

*2) Lexical features*: exploiting the presence of particular term lists such as MeSH, PSI-MI, UMLS, BioLexicon, or in house term lists, or filtering a set of words using stop word lists.

*3) Textual pattern features*: use of particular patterns for expressing protein interactions, some of which had been also applied for finding protein interaction pairs in BioCreative II and II.5 (e.g. 'interacts with', 'binds to').

*4) String features*: using character strings of particular length like strings-of-length-8 tokens used by team 100.

*5) Syntactic features*: using dependency parser output, grammatical patterns or shallow parsing derived phrases as features.

*6) POS tags as features*: selecting words with a particular POS tag as features for a classifier (as done by team 104).

*7) Article metadata features*: metadata provided for each PubMed record (journal, MeSH annotation, author name or affiliation fields).

*8) Semantic features (NER)*: Named entity recognition has been used to identify mentions of genes and proteins, which can be used as features or serve as constraints for grammatical patterns. This feature seemed to be particularly important for specificity.

### IMT

In case of the interaction method task, considerable complexity lies in differentiating between cases where a particular method supports a protein interaction event, as opposed to some other experimental setting where that method is being used, and therefore goes far beyond simple term look-up. Providing more detailed training data in form of representative collections of textual evidence passages or marked method mentions would be important to facilitate further the improvement of performance of text mining tools for associating correctly full text article to interaction detection method terms from a list of 115 potential candidate terms. Under this scenario the overall performance of the best result on the entire set of articles (F-Score 55%, AUC iP/R 35%, MCC 0.54) is quite promising, but also points out that totally automated annotation of interaction methods is not yet solved, and that the resulting strategies would be more appropriate as systems aiding in the manual curation process by suggesting method terms based on highlighting of potential evidence passages. The short time needed by the servers makes it seem reasonable that online, automated systems could be used for this task.

**Figure 5:**
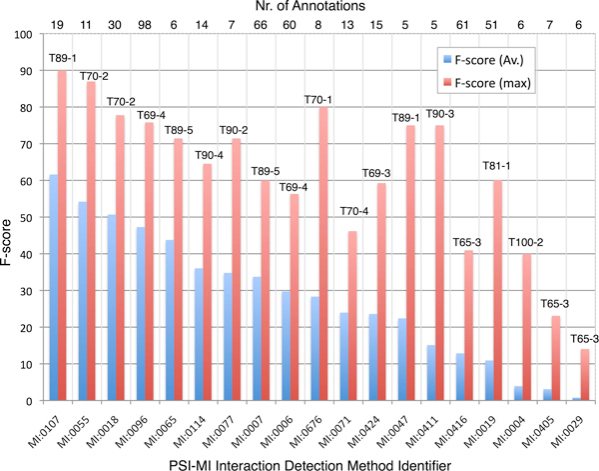
**IMT predictions for relevant method terms**. This figure shows the average F-score (blue) across all runs obtained for test set predictions using PSI-MI interaction detection method terms with at least 5 annotations. Also the best F-score (red) obtained by an individual run is provided.

A considerable fraction of the allowed method terms appear with low frequency in all three data sets. This makes it particularly difficult to detect such methods by supervised methods due to the lack of sufficiently large, representative training data. On the other hand, some methods are more relevant for the curation process and account for a considerable amount of annotations. We therefore carried out a more granular analysis, examining the performance of each run with respect to individual methods, determining those runs (systems) that are most competitive for each of the relevant interaction detection methods (see Figure 5, a more detailed plot on the method distributions is provided in the supplementary material section - additional file 3). When looking at the average F-scores obtained for each method across all runs, it becomes clear that the identification of some methods is more difficult than others. Among the 'easier' methods terms across all runs are MI:0107 (surface plasmon resonance, average F-score of 61.6%), MI:0055 (fluorescent resonance energy transfer, average F-score of 54.2%) and MI:0018 (two hybrid, average F-score of 50.7%). In case of the maximum F-score obtained by participants, the most competitive results were obtained also for MI:0107 (maximum F-score 90.0% by team 89, run 1), followed also by MI:0055 (maximum F-score of 87.0% by team team 70 run 2) and MI:0676 (tandem affinity purification, maximum F-score of 80.0% by team 70, run 1). On the other side, some of the method terms seemed to be especially difficult to detect, including MI:0029 (cosedimentation through density gradient, average F-score of 0.780% and maximum F-score of 14.0%), MI:0405 (competition binding, average F-score of 3.10%, maximum F-score of 23.1%) and MI:0004 (affinity chromatography technology, average F-score of 3.93%, maximum F-score of 40.0%). For all of these three methods, the best runs obtained results that are considerably better than the average. Note that all of these methods are actually types of experiments that can be also mentioned in other contexts not related to PPI characterizations. This implies that they are only supporting PPI experiments under special contextual circumstances. Improvements of predictions for some of the methods are still necessary, but notably for 9 out of the 19 most relevant method terms there was at least one run with an F-score over 70%.

Examining in more detail those strategies used by runs that outperformed other predictions (see Figure 5), we can see that their success heavily depended on the actual (a) interaction method type, (b) the specificity of the method for characterizing PPI versus other experimental settings, and (c) how representative the lexical resources provided by the PSI-MI ontology were for referring to the method in the articles. It is possible to summarize these approaches into the following strategies:

#### 1. Dictionary expansion strategies

##### 1.1 Lexical enrichment by integrating additional terminology based on manual inspection of the training data

This approach was used in case of the best prediction for method MI:0416, where in addition to the official PSI-MI concept 'fluorescence microscopy' and the synonym 'fluorescence imaging' commonly known synonyms were included in the PSI-MI dictionary, such as the term 'immunofluorescence staining'. Some teams also relied on domain experts to add manually additional synonyms based on their background knowledge.

##### 1.2 Lexical enrichment by cross ontology mappings

In order to generate a lexical expansion of the original interaction method ontology, one strategy was manually mapping between PSI-MI terms and MeSH identifiers, using the resulting relations as features to train supervised classifiers. Another approach consisted of first selecting a particular subset of UMLS concept types, and then finding those concepts that shared a name with a term in the PSI-MI ontology and adding the resulting UMLS concept synonyms to the interaction method term dictionary.

##### 1.3 Rule based lexical expansion independent of training data

A range of teams tried to improve the recall of their methods by automatically adding typographical and lexical variants to the original set of method terms, considering alternative use of hyphenation, capitalization, uppercase/lowercase usage, alternative equivalent numeric expressions (arabic, roman and word numerals), substitution by synonymous words, and consideration of acronym and expanded long forms.

#### 2. Feature generation, extraction and selection strategies

##### 2.1 Detection of words, bigrams and collocations associated to ontology terms derived from training data

Another approach to increase the recall of detecting associations between documents and MI ontology terms was based on the initial extraction of n-grams and collocations from the collection of training documents, followed by the calculation of the probability of a method given a particular n-gram or collocation. This strategy seemed to help boosting the results obtained by team 65 in case of the terms MI:0405 (competition binding) and MI:0029 (cosedimentation through density gradient).

##### 2.2 Exploitation of exact and partial word tokens found in the method terms

For many methods there is only a relatively poor association between the exact or partial name of the interaction detection method and whether the name indicates that the detection method was used in a PPI context. Nevertheless in some cases, exact, partial or merged tokens building the method term can be used by machine learning algorithms as features for scoring method-document associations. For instance in case of MI:0006 (anti bait coimmunoprecipitation) and MI:0096 (pull down), team 69 could detect several features with a strong positive correlation to these methods corresponding to both the exact and partial name of the method. In case of MI:0006 positive features included the partial stemmed tokens 'precipit' and 'immunoprecipit' while for MI:0096 the merged token 'pulldown' and the exact token 'pull' show a relatively strong positive association. Team 90 on the other hand exploited the usage of matches between word unigrams and character n-grams (n={2,3,4}) from the PSI-MI definition and synonyms.

##### 2.3 Use of machine learning approaches exploiting features with strong positive or negative correlation with respect to a particular method

Machine learning systems are not only able to exploit tokens forming method terms, but also tokens derived from documents provided as training data in order to predict ontology terms for a given input article. Considering the prediction of team 69 for MI:0424 (protein kinase assay), the strongest notable positive features are the stemmed tokens 'vitro', 'phosphoryl', 'kinase' and 'juxtamembrane'. The token 'kinase' is the only feature that relates to the name of the detection method, and all but 'juxtamembrane' are relatively common terms. The only notable negative features for this interaction method are the stemmed tokens 'monom' and 'migrat', which are also relatively common terms. These results also show that sometimes the significance of some features is not particularly transparent in terms of human interpretation.

#### 3. Pattern matching and rule based approaches

Capturing variations of interaction method expressions can be addressed by using regular expression matches and heuristics. In case of team 100, their prediction for MI:0004 (affinity chromatography technology) required that both their pattern-matching and kNN method generated a positive hit. In case of the pattern-based approach a set of additional tokens were required to be not co-mentioned in order to distinguish this term from MI:0676 (tandem affinity purification). Their pattern for extracting MI:0004 was:

IF sentence contains "affinity" AND "chromatography" AND "purification"

      IF sentence contains "tandem" OR "tap"

            assign MI:0676            ELSE            assign MI:0004

Our database curator collaborators have suggested that it could be meaningful to carry out a grouping of equivalent methods that are experimentally related. For instance the terms MI:0006 (anti bait coimmunoprecipitation), MI:0007 (anti tag coimmunoprecipitation), MI:0019 (coimmunoprecipitation), MI:0858 (immunodepleted coimmunoprecipitation) and MI:0676 (tandem affinity purification) could be grouped under the term MI:0004 (affinity chromatography technology) for analysis purposes, or MI:0028 (cosedimentation in solution) and MI:0029 (cosedimentation through density gradient) could be grouped under MI:0027 (cosedimentation). Unfortunately, the ontological structure of PSI-MI itself does not directly provide sensible groupings and it would require extensive manual classification and discussions among different curators to generate a proper consensus on accurate method groupings.

## Conclusions

The PPI tasks of BioCreative III tried to address relevant aspects for both database curators as well as general biologists interested in improving the retrieval of interaction relevant articles and association of ontology terms and experimental techniques to full text papers. Large training, development and test set collections were provided to participating teams and these publicly available corpora should represent a valuable resource for future implementations and evaluations of biomedical text mining. From the results obtained, it seems that classification of PPI relevant abstracts using participating systems is able to improve the selection of relevant articles for database curators and biologists, both in terms of number of items that need to be reviewed as well as in terms of time saving. In order to derive practically useful applications from this task, the systems need to be at least accessible online. Combining the different runs for the ACT resulted in a consensus system with better performance than the best individual run, an aspect that already motivated the implementation of the BioCreative Meta-Server infrastructure [40]. We presented a detailed analysis of curator classification times and agreement between human annotators for the article classification task, which allows to estimate better the theoretical performance limit of text mining systems. It remains for the future to carry out this type of analysis in more complex scenarios, for instance, based on the individual steps followed in biological annotation workflows like the one used by interaction databases, and quantify the effect of integrating text mining modules in terms of curation efficiency measured in time units or annotation records over some baseline manual annotation. To focus in the future on particular types of interactions such as phosphorylation relations, or particular protein functional types (kinases or phosphatases) could be interesting for data consumers.

In summary, current state-of-the-art systems are likely to have a significant impact on simplifying (but not completely automating) the manual process of article selection and could potentially be adapted not only to score individual articles, but also to determine the most relevant journals for each biocuration type. The initial setting of this task had to be slightly modified to make resulting systems more practically relevant. Analyzing records from one month of PubMed abstracts with links to free full text articles (which can be considered the first level approach) resulted in a collection that only covered a minor fraction of PPI relevant journals. Far less than 5% of the records were PPI relevant in general, and even a smaller set was PPI annotation relevant, as most articles are related to the clinical domain.

In case of the IMT, participating systems did significantly better than a baseline term-lookup approach. The main difficulties for this second task arise from the many different ways of describing a given experimental method, handling PDF articles, and the heterogeneous journal composition. From the analysis of strategies used for the IMT and the performance obtained for individual methods, it seemed that certain techniques were more efficient for certain terms, which makes sense under the assumptions that some of the terms can be better identified using pattern based approaches, while others can be better detected using machine learning or using some sort of expanded lexical resources. Moreover, some method terms are very general and can be used in other contexts that are not PPI relevant. Note that a number of method terms/acronyms are also highly ambiguous (e.g. '2H' or 'CD'). Providing more complete lexical resources of method terms by the PSI-MI ontology developers could also have a positive impact on automated systems. Another challenging aspect, even for database curators, is the complexity in mapping annotations to the right granularity of terms in the ontology. The distances in the ontological structure can not be used to produce meaningful scores for calculating semantic similarities between terms in the PSI-MI ontology. A proposal to overcome this issue is based on grouping method concepts together that are equivalent in terms of annotation purposes. This interesting strategy of a more coarse-level annotation nonetheless requires a considerable manual effort in deriving such method groupings, which illustrates that having evidence passages derived from the text for the annotations would aid the human interpretation of the assigned experimental qualifiers for PPIs. Table 7 provides an overview of external tools used by participating teams.

**Table 7:** Overview of tools and resources. Collection of external tools and resources used for the PPI tasks by participating teams.

Name	Type	URL	Summary
MALLET	ML	[48]	Framework for feature extraction, logistic regression models and inference

SVMPerf	ML	[83]	Support Vector Machine software for optimizing multivariate performance measures

Weka	ML	[64]	Collection of machine learning algorithms for data mining, useful for feature selection

LIBSVM	ML	[84]	Software for support vector classification

Matlab	ML	[85]	Data analysis, and numeric computation software

Liblinear	ML	[86]	Linear classifier software

MEGAM	ML	[87]	Software for maximum entropy model implementation

C&C CCG parser	NLP	[55]	Parser and taggers are written in C++

TreeTagger	NLP	[88]	Part-of-speech tagger (trained on PENN treebank)

SNOWBALL	NLP	[89]	Stemming program

NooJ	NLP	[90]	Corpus processing and dictionary matching

Lucene	NLP	[53]	Full-featured text search engine library

LingPipe	NLP	[91]	Tool kit for processing text using computational linguistics

PSI-MI	Lexical	[46]	Molecular Interaction Ontology used by PPI databases

UMLS	Lexical	[47]	Unified Medical Language System which contains a large vocabulary database about biomedical and health-related concepts

MeSH	Lexical	[92]	Vocabulary thesaurus used for indexing PubMed

ChEBI	Lexical	[93]	Chemical Entities of Biological Interest

BioLexicon	Lexical	[52]	Terminological resources integrating data from various bioinformatics collections

Stop words	Lexical	[44]	Collection of words that are filtered out prior to processing of natural language data

NLProt	BioNLP	[94]	SVM-based tool for recognition of protein-names in text

OSCAR3	BioNLP	[95]	Tool for recognition of chemical name mentions in text

ABNER	BioNLP	[60]	Bio-Named entity recognition (proteins, genes, DNA, etc.)

Performance will certainly increase with the amount of readily available training data and as more interest in this particular area of entity types is raised. Further improvements of the task data settings would require to provide participating teams with data at the level of large representative collections of support textual statements (e.g. individual evidence sentences) and true negative statements, in addition to a better set of synonyms for the concepts, both of which could in principle be provided as byproduct of the biocuration process. Another option for future settings would be to distinguish between closed and open predictions, i.e. those that only use the provided training data and those that make use of external resources for generating their predictions respectively, something that had been considered in BioCreative I [74]. Carrying out the article classification task on the same collection of abstracts vs. full text articles could illustrate the advantage of using one document type versus the other.

On the positive side, the relatively good performance (with respect to the global results) of the online team (89) combined with their very competitive server annotation times (3.7 sec/article) clearly demonstrates that online, high-quality BioNLP can be implemented in ways where processing times are acceptable to serve end-users on demand. Other systems were also able to provide predictions in a competitive time frame, for instance for the ACT, team 104 completed the predictions in 1.29 seconds, while team 89 required only 2 seconds per article. In case of the IMT, predictions took on average 120 seconds per full text article for team 69, but their system is designed to allow batch processing on a multi-CPU server which would improve their efficiency. Team 89 needed approximately 4 seconds per article for the same task.

The PPI databases BioGRID and MINT contributed to the data preparation of BCIII with the aim of promoting the development of more efficient text-mining tools that, taking advantage of ad hoc curated datasets and databases, would in return result in systems that could assist their biocuration workflows. The development of such tools will be critical for the future of biological databases to keep up the pace with information published in the literature. Text-mining tools should thus be able to help in the selection of the relevant literature and in the annotation process itself. It is also clear that successful integration of such systems into database annotation pipelines can be achieved only by close collaboration with biological databases, tuning the systems towards particular specifications demanded by curators, ultimately serving the general biological community in terms of improved information access. In case of the PPI tasks of BioCreative III, it would be valuable for biologist end users to have a customizable e-mail alert system of PPI relevant abstracts for user specified queries or journals as well as a system that allows uploading PDF full text articles, returning a ranked list of interaction detection methods together with evidence passages. A closer examination of the evidence sentences provided by participating teams for the IMT would be interesting in the future, in combination with some highlighting within the article context to make human interpretation easier.

## Competing interests

The authors declare that they have no competing interests.

## Authors' contributions

MK, MV and FL organized the task, prepared the data collections and the manuscript, and carried out the evaluation. They should be considered all as first authors. DG in collaboration with MK has designed and built the MyMiner application used for the ACT. AV supervised and coordinated this task. Two participants from each team contributed with a systems description, revised the manuscript and provided input. All authors read and approved the final maunscript.

## Supplementary Material

Additional file 1 **ACT annotation guidelines**. Basic classification criteria for PPI abstracts. Click here for file

Additional file 4 Evaluation metrics overview. Details on the calculation of the used evaluation scores. Click here for file

Additional file 2 **ACT example run**. iP/R curve of the best team (73, S. Kim and W. J. Wilbur) in the Article Classification Task. Circle 1: Of the top 2% (130) of all results, approx. 90% (120) are relevant abstracts. Circle 2: To find half (295) of all relevant abstracts (Recall around 50%), a human going over the ranked list only has to look at the first 7% (421) of all results; and approx. 2/3 (Precision around 70%) of those abstracts will be relevant. Click here for file

Additional file 3 **IMT method distribution**. Distribution of interaction detection methods across the different IMT data sets. Click here for file
