# Supplementary material for: The Protein-Protein Interaction tasks of BioCreative III: classification/ranking of articles and linking bio-ontology concepts to full text
Source: BMC Bioinformatics. 2011 Oct 3;12(Suppl 8):S3. doi: 10.1186/1471-2105-12-S8-S3 (PMC3269938; doi:10.1186/1471-2105-12-S8-S3)
Supplement: Additional file 1 — ACT annotation guidelines. Basic classification criteria for PPI abstracts. [file 1471-2105-12-S8-S3-S1.zip › additional1/GenProt_PPI.htm]

Home


|  |
| --- |
| Summary  The aim of this annotation task is to classify abstracts into Protein-Protein Interaction (PPI) relevant and non-relevant cases. Although the only way to be totally sure if an article is PPI annotation relevant is by manually revising the full text article, abstracts and titles can still contain enough clues/information to decide if revision of the full text is worthwhile in many cases. The result of the manual classification should result in a training and test set that can be used to develop and evaluate systems that can classify and rank (prioritize) article for PPI relevance. Such a system could be useful as:   (1) a periodical alert system for PPI relevant articles recently published (e.g. last week, month).   (2) to rank articles relevant for PPI based on some keyword search (e.g. phosphatases)     The manual classification will be done using the MyMiner system 'file Labeling' option.     The titles and abstracts should be manually revised to decide if a record is PPI relevant. Highlighting with positive and negative terms can help in the manual classification, but it is still necessary to cross check by reading if the record is relevant or not.      How to use MyMiner   There is an online tutorial how to use the MyMiner system. The input file must be a tab separated file (three columns: Id, title, abstract). You have to:     1. Click on the File Labeling option  2. Select and upload the file of interest (i.e. the corpus/abstract collection to be labeled)  3. Write label 1 (i.e. 'Not' stands for not Protein-Protein Interaction relevant).  4. Click on Add new label (above the field): define the labels you will use  5. Write label 2 (i.e. 'PPI', stands for Protein-Protein Interaction relevant).  6. Click on GO!  7. You should see now the abstract to be labeled  8. You can enter positive or negative words to be highlighted (then press the GO! to show these labels)  9. Read the text to determine whether the abstract/title is relevant or not for PPI: click on the corresponding labels (PPI or Not)  10. Then classify the next case, and so on.  11. Then classify the next case, and so on.     Note that the time needed to classify each record is recorded, if you want to pause or stop for a while please Click on the red circle on the left lower corner (Export tagged file) to avoid outliers in the classification time. With this option the curated file and the missing abstracts are downloaded to your PC. To continue the classification, you would need to upload this generated file. Try to save the annotation always after some time to avoid that your curation is lost due to timeout session and connection error!!! |
