# Supplementary material for: The Protein-Protein Interaction tasks of BioCreative III: classification/ranking of articles and linking bio-ontology concepts to full text
Source: BMC Bioinformatics. 2011 Oct 3;12(Suppl 8):S3. doi: 10.1186/1471-2105-12-S8-S3 (PMC3269938; doi:10.1186/1471-2105-12-S8-S3)
Supplement: Additional file 1 — ACT annotation guidelines. Basic classification criteria for PPI abstracts. [file 1471-2105-12-S8-S3-S1.zip › additional1/GenProt_PPI_files/page0001.htm]

PPI RELEVANT


|  |
| --- |
| Criteria for classifying as PPI relevant                                                                                                                                                                                                                                                                                                                                                                                       Note: If the curator is not sure if the entity is actually a protein, he can consult external resources, databases and the web to disambiguate those cases. |

|  |  |
| --- | --- |
| 1. | Protein interaction in title or abstract |
| 2. | Articles characterizing a protein interaction |
| 3. | Methods that indubitably are used for the detection of protein interactions (coprecipitate, pulled-down, 2-hybrid) |
| 4. | Transient interactions, e.g. phosphorylation/dephosphorylation events are relevant |
| 5. | Phosphorylation type of interactions are only relevant if it is clear, or there is some evidence in the paper that the actor and patient of the phosphorylation relation is described/ characterized |
| 6. | Autophosphorylation events are relevant |
| 7. | Homomeric, heteromeric, homomultimeric (such as dimeric, trimeric, tetrameric, pentameric) interactions are relevant |
| 8. | Interactions characterized in vivo or in vitro are relevant |
| 9. | Co-localization of proteins are relevant |
| 10. | Interactions of mutant or modified proteins with other proteins are relevant |
| 11. | Cofractionation of two proteins are relevant |
| 12. | Enzyme and processed substrate if protein then such interactions are relevant |
| 13. | Also there are cases of protein-protein complexes which additionally interact also with DNA, such cases are relevant if there is a identifiable protein-protein interaction present, regardless if there are other additional binding partners that are for instance other molecules. An example case would be a transcription factor complex of two proteins , lets say protein A and protein B, which as a complex bind to a promoter |
| 14. | Protein interaction with toxins or ligands, when they are protein moiety |
| 15. | Protein ligand and its interactions with receptor are relevant |
| 16. | Ubiquitination, Sumoylation of protein are relevant |
|  |  |
|  |  |
|  |  |
| Example cases of PPI relevant statements | |
| 1. | MeCP2 cofractionated with Brm |
| 2. | MeCP2 associates in vivo with the Brm containing SWI/SNF complex |
| 3. | MeCP2 was present in immunoprecipitates of antibody to Brm |
| 4. | MeCP2 was copurified from Brm isolates |
| 5. | (GST) pull-down experiments using bacterially expressed MeCP2 and in vitro−translated recombinant SWI/SNF subunits |
| 6. | MeCP2 specifically interacted with Brm2 |
| 7. | we carried out reciprocal immunopurifications using glycerol-gradient fractions 17−20 followed by western-blot analysis using antibodies to MeCP2 and Brm |
| 8. | Results of these analyses are consistent with the existence of a complex containing MeCP2, Brm and BAF57 |
| 9. | Antibody to Brm immunoprecipitated MeCP2 from soluble cross-linked protein extracts |
| 10. | Staining for the protein determinants showed considerable, but not precise, overlap for Brm and HDAC2 and colocalization of Brm and MeCP2 |
| 11. | ARR3 was found to interact with At5G21510 |
| 12. | eE2 is properly folded, as determined by binding to human CD81 |
