# Supplementary material for: The Protein-Protein Interaction tasks of BioCreative III: classification/ranking of articles and linking bio-ontology concepts to full text
Source: BMC Bioinformatics. 2011 Oct 3;12(Suppl 8):S3. doi: 10.1186/1471-2105-12-S8-S3 (PMC3269938; doi:10.1186/1471-2105-12-S8-S3)
Supplement: Additional file 1 — ACT annotation guidelines. Basic classification criteria for PPI abstracts. [file 1471-2105-12-S8-S3-S1.zip › additional1/GenProt_PPI_files/page0002.htm]

PPI IRRELEVANT


|  |
| --- |
| Criteria for classifying as NOT-PPI relevant                                                                                                                                                                                         Note: If the curator is not sure if the entity is actually a protein, he can consult external resources, databases and the web to disambiguate those cases. |

|  |  |
| --- | --- |
| 1. | Interactions of protein with chemical compounds, macromolecular structures, cell lines, cell types etc |
| 2. | Genetic / gene regulation interactions |
| 3. | Induction / inhibition of gene expression |
| 4. | Protein-promoter binding |
| 5. | Protein-Lipid binding are non-relevant (19414018) |
| 6. | RNA - protein interactions |
| 7. | DNA - protein interactions |
| 8. | Protein-membrane interaction |
| 9. | Host-pathogen interactions (e.g. interaction of a virus as a whole with some host cell) |
| 10. | Protein interactions implicit in the name of a protein are considered as non relevant, as no interaction event needs to be characterized in the article. |
| 11. | Aberrant fusion proteins that result from translocations are not relevant (e.g 19718047 ) |
| 12. | Mention of a general protein complex (e.g. nuclear pore complexes (NPCs)) without specifying some of its individual protein components is non-relevant |
| 13. | Interactions of some synthetic peptide (drug) that can not me associated to any real protein are not relevant |
| 14. | Interactions of the N-terminal and C-terminal part of the SAME protein (intra-protein interactions) are non-relevant, if the interaction is between two DIFFERENT proteins then it is relevant |
| 15. | Regulatory interactions that are not implying physical interactions (e.g. up-regulation, general induction) are non-relevant: only if physical interaction is clear from the context then the interaction is relevant |
| 16. | A computer tool that predicts protein interactions is not relevant |
| 17. | Pathway interactions are considered as irrelevant |
| 18. | Synthetic peptide interaction is considered to be irrelevant |
| 19. | Protein interactions not directly described in the article. Eg. Signaling pathway, downstream reactions and pathway interaction that are not direct are irrelevant |
| 20. | General binding mechanisms such as capping of proteins (Eg.19556544) |
| 21. | In vitro antigen-antibody reactions, eg using monoclonal antibodies or recombinant antibodies |
| 22. | A general events such as protein cleavage, apoptosis, phosphorylation without mentioning the protein that process these events |
| 23. | Transcription activation by proteins |
| 24. | GFP (Green Fluorescent protein) or it mutant form (e.g YFP) tagged with other protein for the localization of target protein, or used as reporter or biosensor are not relevant |
|  |  |
|  |  |
|  |  |
| Example cases of NOT-PPI relevant statements | |
| 1. | HIP-55 is an SH3-binding protein important in T-cell receptor signalling. (Comment: It states that the protein binds to certain domains in general but does not mention any concrete protein) |
| 2. | Treatment is limited, and efficacy depends upon the infecting strain and the initial viral load. The HCV envelope glycoproteins (E1 and E2) are involved in receptor binding, virus-cell fusion, and entry into the host cell. (Comment: too general statement) |
| 3. | GF2 is first recruited to the inner leaflet of plasma membranes, in a process that is mediated by the phosphoinositide PtdIns(4,5)P(2). (Comment: interaction with a compound not a protein) |
| 4. | Ant-plant interactions represent a diversity of strategies, from exploitative to mutualistic, and how these strategies evolve is poorly understood. (Comment: interaction between to species not proteins) |
|  |  |
