# Supplementary material for: The Protein-Protein Interaction tasks of BioCreative III: classification/ranking of articles and linking bio-ontology concepts to full text
Source: BMC Bioinformatics. 2011 Oct 3;12(Suppl 8):S3. doi: 10.1186/1471-2105-12-S8-S3 (PMC3269938; doi:10.1186/1471-2105-12-S8-S3)
Supplement: Additional file 1 — ACT annotation guidelines. Basic classification criteria for PPI abstracts. [file 1471-2105-12-S8-S3-S1.zip › additional1/GenProt_PPI_files/page0003.htm]

POSITIVE KEYWORDS


|  |
| --- |
| POSITIVE KEYWORDS |

|  |
| --- |
| affinity chromatography |
| activates |
| is a target of |
| dimerization |
| interacts biochemically with |
| bound |
| autophosphorylation |
| activated |
| cleaved by |
| directly phosphorylates |
| substrate |
| interact physically |
| component of the |
| homodimer |
| dimer |
| dimer formation |
| binds to the |
| colocalizes |
| associated with |
| specific recognition of |
| binding sites |
| heterocomplexes |
| pull-down |
| dimers |
| co-immunoprecipitation |
| colocalization |
| yeast two-hybrid |
| interacted |
| interact with |
| assembly |
| proteomic screen |
| interacting |
| dephosphorylates |
| activation of |
| receptor interactions |
| interaction |
| by binding to |
| by inhibiting |
| Dimerization |
| phosphorylation |
| homotrimeric |
| immunocolocalization |
| pentameric |
| tetramer |
| reciprocal coimmunoprecipitations |
| hexameric |
| homohexamer |
| homodimer |
| binding |
| interacts with |
| dimeric |
| inhibits |
| bind |
| complex |
| co-depletion |
| colocalize |
| inhibition of |
| ubiquitinates |
| ubiquitination |
| targets |
| associates tightly with |
| homododecameric |
| phosphorylate |
| trimer |
| protein |
| fused |
| fusion |
| association |
| stabilizes |
| direct |
| homododecameric |
| phosphorylate |
| trimer |
| formation |
| form |
| domain |
| motif |
| protein |
|  |
