# Supplementary material for: The Protein-Protein Interaction tasks of BioCreative III: classification/ranking of articles and linking bio-ontology concepts to full text
Source: BMC Bioinformatics. 2011 Oct 3;12(Suppl 8):S3. doi: 10.1186/1471-2105-12-S8-S3 (PMC3269938; doi:10.1186/1471-2105-12-S8-S3)
Supplement: Additional file 1 — ACT annotation guidelines. Basic classification criteria for PPI abstracts. [file 1471-2105-12-S8-S3-S1.zip › additional1/GenProt_PPI_files/page0004.htm]

NEGATIVE KEYWORDS


|  |
| --- |
| therapeutic |
| speech |
| fetal |
| topographic |
| Ukraine |
| microarray |
| researcher |
| dictionary |
| data-server |
| Russia |
| Romania |
| Macedonia |
| Poland |
| Hungary |
| ontology |
| knowledge |
| annotation |
| curator |
| Belarus |
| mining |
| KEGG |
| MicroRNAs |
| hepatectomy |
| August |
| genome-wide |
| China |
| vaccine |
| alcohol use |
| Solar System |
| feelings |
| emotion |
| cognitive |
| emotional |
| nightsides |
| drinker |
| planet |
| extrasolar |
| Netherlands |
| primary care |
| colonization |
| Bayesian method |
| villager |
| pathogenicity |
| Odor |
| days |
| diarrhea |
| pretreated |
| alexithymia |
| alexithymic |
| diagnosing |
| Vienna |
| exposure |
| personality |
| Empathy |
| week |
| symptoms |
| influx |
| serotype |
| Gabon |
| deaths |
| annually |
| nonmate |
| survival |
| cancer risk |
| hyaluronan |
| males |
| cancer risk |
| polyps |
| guidelines |
| biopsy |
| epilepsy |
| SCOP |
| vicarious |
| stranger |
| Pharmacologic |
| pharmacoresistant |
| specimen |
| lesions |
| antipsychotic |
| Lipid raft |
| visibility |
| encyclopedia |
| visual |
| hominids |
| radiological |
| enrolled |
| ORIs |
| higher risk |
| young |
| intelligence |
| anatomical |
| tractography |
| drinking |
| vertical transmission |
| genetic background |
| intracerebral |
| cerebral activity |
| Musical |
| consciousness |
| carotid artery |
| hyperlipidemic |
| knockout |
| German |
| subcortical |
| devastating |
| Somatic |
| prognostic |
| anesthetic |
| polymer |
| cortical |
| perceptual |
| metabolites |
| neurons |
| death |
| anesthesia |
| relatives |
| pathophysiology |
| learning |
| game |
| hydrodynamically |
| reinforcement |
| injected |
| alloreactive |
| immune response |
| mother |

|  |
| --- |
| child |
| reflux |
| animals |
| obesity |
| dietary |
| genotype |
| deficiency |
| infection |
| Venezuela |
| biofluids |
| fever |
| susceptible |
| epidemic |
| vegetarian |
| women |
| prospective |
| meat |
| photopheresis |
| Blood |
| Oral |
| incidence |
| linkage disequilibrium |
| individuals |
| case-control |
| xenograft |
| age and sex |
| ethnic |
| susceptibility |
| feeding |
| eater |
| British |
| mosquitoes |
| decision trees |
| progression |
| immunotherapies |
| handicap |
| transgenic mice |
| infiltrate |
| pathologist |
| aetiology |
| people |
| working-aged |
| adversities |
| sex-adjusted |
| patient |
| smoking |
| cholesterol |
| SNP array |
| dosage |
| hypertension |
| blood pressure |
| stroke |
| diastolic |
| Washington |
| systolic |
| insulin |
| antidiabetic |
| heavy drinking |
| questionnaire |
| Health |
| Social |
| tomography |
| spatiotemporal |
| father |
| paternal |
| germ-line |
| birth |
| promoter |
| chromatin modification |
| cognition |
| body temperature |
| Africa |
| phase III trial |
| India |
| Japanese |
| menopausal |
| middle-aged |
| Middle East |
| Mediterranean |
| Cyprus |
| Malaysia |
| Europe |
| Greece |
| Spain |
| immunization |
| administered |
| pathological |
| quantitative trait loci |
| ulcer |
| engraftment |
| epigenetic |
| Cardiac |
| arrhythmogenic |
| Mexico |
| fisheries |
| diagnostics |
| countries |
| convergence |
| ecological |
| Genomic |
| California |
| ancestral |
| Markov |
| hidden Markov model |
| QTL |
| radiotherapy |
| surgery |
| esophagitis |
| retrospective |
| hypermethylation |
| hemorrhage |
| embolization |
| CGH |
| Indian |
| Hispanic |
| inflammation |
| comparative genomic |
| Chinese |
| HapMap |
| Singapore |
| smoke |
| biopsies |
| Monte Carlo |
| hematoma |
| case report |

|  |
| --- |
| transcatheter |
| hospital |
| infant |
| Beijing |
| healthy |
| database |
| temporal |
| perception |
| compute |
| industries |
| symbiotic |
| GeneChip |
| cost-effective |
| prevalence |
| Hungarian |
| cohort |
| diagnosis |
| spelling |
| neural network |
| voxels |
| memory |
| malabsorption |
| forestry |
| Computer |
| agriculture |
| economic |
| year |
| old |
| wildlife |
| worldwide |
| global |
| American |
| morbidity |
| follow-up |
| subcutaneous |
| prognosis |
| surveillance |
| month |
| refractory |
| endoscopic |
| donor |
| knowledge base |
| Python interpreter |
| scripting |
| bioinformatic |
| fear |
| national |
| Canada |
| myopia |
| amygdala |
| autoimmunity |
| Biliary |
| management |
| Antioxidants |
| United States |
| ginseng |
| friendship |
| mobile phone |
| satisfaction |
| web-based |
| Darwin |
| botanist |
| Biomechanic |
| America |
| South |
| Asia |
| Southeast |
| behavioral |
| formalin |
| paraffin |
| tissue |
| illumination |
| voltage clamp |
| depolarization |
| female |
| pedigree |
| generations |
| Illumina |
| Solexa |
| patients |
| treatment |
| epidemiologic |
| children |
| adults |
| subjects |
| chronic |
| transplantation |
| adult |
| woman |
| orthotopic |
| childhood |
| computation |
| transcriptomic |
| genome |
| simulation |
| castration |
| clinical |
| antiretroviral |
| drug |
| postnatal |
| prenatal |
| histories |
| reproductive |
| ovarian |
| nutritional |
| Pregnant |
| pubertal |
| offspring |
| diet |
| maternal |
| caloric |
| regenerative |
| transplanted |
| preclinical |
| outbreed |
| somatosensory |
| regimes |
| symptomatic |
| orphan |
| neurological |
| Food |
| double-blind |
| randomized |
| trials |
| placebo |

|  |
| --- |
| blinding |
| randomization |
| salinity |
| stress |
| productivity |
| mentalizing |
| gestural |
| Communication |
| Spoken |
| language |
| mental |
| produce |
| gestures |
| semantic |
| supercooled |
| glass |
| Einstein |
| anemia |
| hours |
| investment |
| mutualist |
| evolution |
| herbivore |
| monophyletic |
| phylogenetic |
| colonize |
| parasites |
| paleogenetics |
| bioinformatics� |
| algorithm |
| linguistics |
| grammar |
| programming |
| parsing |
| phylogeny |
| maximum likelihood |
| probabilistic |
| phylogenies |
| population |
| Spanish |
| Italian |
| GWAS |
| electrophysiological |
| behaviour |
| supernova |
| cosmic |
| Galactic |
| century |
| computing |
| illnesses |
| respiratory |
| airway |
| behavior |
| nasopharyngeal |
| web server |
| RNA |
| DNA |
| allele |
| genes |
| DNA |
| RNA |

|  |
| --- |
| NEGATIVE KEYWORDS |
