# Supplementary material for: The Protein-Protein Interaction tasks of BioCreative III: classification/ranking of articles and linking bio-ontology concepts to full text
Source: BMC Bioinformatics. 2011 Oct 3;12(Suppl 8):S3. doi: 10.1186/1471-2105-12-S8-S3 (PMC3269938; doi:10.1186/1471-2105-12-S8-S3)
Supplement: Additional file 1 — ACT annotation guidelines. Basic classification criteria for PPI abstracts. [file 1471-2105-12-S8-S3-S1.zip › additional1/GenProt_PPI_files/punused.htm]

Home | About Us | Contact Us | Service List | Project List

Home | About Us | Contact Us | Service List | Project List

Home | About Us | Contact Us | Service List | Project List

Home | About Us | Contact Us | Service List | Project List

Home | About Us | Contact Us | Service List | Project List

Home | About Us | Contact Us | Service List | Project List

Home | About Us | Contact Us | Service List | Project List

Home | About Us | Contact Us | Service List | Project List

Home | About Us | Contact Us | Service List | Project List
