# Supplementary material for: The Protein-Protein Interaction tasks of BioCreative III: classification/ranking of articles and linking bio-ontology concepts to full text
Source: BMC Bioinformatics. 2011 Oct 3;12(Suppl 8):S3. doi: 10.1186/1471-2105-12-S8-S3 (PMC3269938; doi:10.1186/1471-2105-12-S8-S3)
Supplement: Additional file 4 — Evaluation metrics overview. Details on the calculation of the used evaluation scores. [file 1471-2105-12-S8-S3-S4.pdf]

## BioCreative III – PPI Task evaluation metrics detailed description

For BioCreative III, the exact same evaluation measures as for BioCreative II.5 were used (see Leitner et al, IEEE/ACM Trans Comput Biol Bioinform. 2010 7(3):385-99). Four major evaluation metrics were used, namely MCC score (Matthew's Correlation Coefficient), F-measure, Accuracy, and AUC iP/R (Area Under the interpolated Precision/Recall Curve). In all following formulas, the following variables are used: TP: count of true positive predictions; FP: count of false positive predictions; TN: count of true negative predictions; FN: count of false negative predictions. The MCC score then is defined as:

$$MCC = \sqrt{\frac{\chi^2}{n}} = \frac{TP*TN - FP*FN}{\sqrt{(TP+FP)(TP+FN)(TN+FP)(TN+FN)}}$$

(1)

As the MCC score is also known as the Phi coefficient, this relationship is shown in (1) via the Chi-square statistic. The major advantage of the MCC is that is unbiased by any set disequilibrium between true and false classes. The F-measure is well known and defined as:

$$F_\beta = \frac{(1+\beta^2)*(p*r)}{\beta^2*(p+r)}$$

(2)

It represents the harmonic mean between precision (p = TP / (TP+FP)) and recall (r = TP / (TP+FN)) when the parameter β = 1, as used for the challenge. It is less robust against unbalanced sets, but as it is very common in NLP, the metric is added to compare to other evaluations. The third measure used was Accuracy, defined as:

$$Acc = \frac{TP+TN}{TP+FP+FN+TN}$$

(3)

This measure defines how close a result is to the true classification. As with F-measure, it even more biased by unequal sets, however. It, too, was added because it is used traditionally in many evaluations. Last, the AUC iP/R is defined as follows:

$$A(f_{pr}) = \sum_{j=1}^{n}(p_{i_j}*(r_j - r_{j-1}))$$

(4)

Where $r_j$ is the recall at $p_{ij}$ (interpolated precision), defined as:

$$p_i(r) = \max_{r' \geq r} p(r')$$

(5)

AUC iP/R has been explained in detail in many text books and publications, such as recently in Manning et al, Introduction to Information Retrieval, (Cambridge University Press, 2008). In a nutshell, it is a measure for the quality of the ranking of an ordered result set. This is particularly important for the evaluation of the results with respect to their usability for humans, assuming they would be presented with the classification results ordered from the most to the least likely prediction.
